# Identification of a key locus, *qNL3.1*, associated with seed germination under salt stress via a genome-wide association study in rice

**DOI:** 10.1007/s00122-023-04252-x

**Published:** 2023-03-13

**Authors:** Chengfang Zhan, Peiwen Zhu, Yongji Chen, Xinyi Chen, Kexin Liu, Shanshan Chen, Jiaxiao Hu, Ying He, Ting Xie, Shasha Luo, Zeyuan Yang, Sunlu Chen, Haijuan Tang, Hongsheng Zhang, Jinping Cheng

**Affiliations:** 1 National Key Laboratory of Crop Genetics & Germplasm Enhancement and Utilization, Jiangsu Collaborative Innovation Center for Modern Crop Production, Hainan Yazhou Bay Seed Lab, Jiangsu Province Engineering Research Center of Seed Industry Science and Technology, Nanjing Agricultural University, Nanjing, China; 2 State Key Laboratory of Rice Biology & Ministry of Agricultural and Rural Affairs Laboratory of Molecular Biology of Crop Pathogens and Insects, Zhejiang University, Hangzhou, 310058 China

## Abstract

**Key message:**

Two causal*** OsTTL* and* OsSAPK1*** **genes of the key locus* qNL3.1* significantly associated with seed germination under salt stress were identified via a genome-wide association study, which could improve rice seed germination under salt stress.**

**Abstract:**

Rice is a salt-sensitive crop, and its seed germination determines subsequent seedling establishment and yields. In this study, 168 accessions were investigated for the genetic control of seed germination under salt stress based on the germination rate (GR), germination index (GI), time at which 50% germination was achieved (*T*_50_) and mean level (ML). Extensive natural variation in seed germination was observed among accessions under salt stress. Correlation analysis showed significantly positive correlations among GR, GI and ML and a negative correlation with *T*_50_ during seed germination under salt stress. Forty-nine loci significantly associated with seed germination under salt stress were identified, and seven of these were identified in both years. By comparison, 16 loci were colocated with the previous QTLs, and the remaining 33 loci might be novel. *qNL3.1*, colocated with *qLTG-3*, was simultaneously identified with the four indices in two years and might be a key locus for seed germination under salt stress. Analysis of candidate genes showed that two genes, the similar to transthyretin-like protein *OsTTL* and the serine/threonine protein kinase *OsSAPK1*, were the causal genes of *qNL3.1*. Germination tests indicated that both *Osttl* and *Ossapk1* mutants significantly reduced seed germination under salt stress compared to the wild type. Haplotype analysis showed that Hap.1 of *OsTTL* and Hap.1 of *OsSAPK1* genes were excellent alleles, and their combination resulted in high seed germination under salt stress. Eight accessions with elite performance of seed germination under salt stress were identified, which could improve rice seed germination under salt stress.

**Supplementary Information:**

The online version contains supplementary material available at 10.1007/s00122-023-04252-x.

## Introduction

Soil salinity is one of the most significant abiotic stresses in plants. It is estimated that over 6% of the world’s total land area and 20% of irrigated land are affected by salt stress (Munns and Tester 2008). Rice (*Oryza sativa* L.) is an important food crop worldwide. The frequent occurrence of soil salinity has been identified as the most widespread soil problem in rice production. Rice is rated as a salt-sensitive crop (Ahmadi et al. 2011). Soil salinity inhibits seed germination and seedling establishment, hampers plant growth throughout the whole growth stage and ultimately reduces crop yield in fields. Seed germination is a vital phase in the plant life cycle that determines subsequent seedling establishment and crop yield (Rajjou et al. 2012). Moreover, the direct seeding of rice has become increasingly popular in many Asian countries due to the lower labor demand and the operational simplicity (Kumar and Ladha 2011; Liu et al. 2015). Therefore, the improvement in seed germination under salt stress becomes more important in rice breeding.

Seed germination under salt stress is a complex quantitative trait and is regulated by polygenes. In *Arabidopsis thaliana*, the zinc finger-containing glycine-rich RNA-binding protein *atRZ-1a*, has a negative impact on seed germination under salt stress (Kim et al. 2007). Mitochondrial thioredoxin-o (*AtTrxo1*), which is transcriptionally regulated by the basic leucine zipper *AtbZIP9* and the zinc finger protein *AtAZF2*, affects seed germination under salt stress (Ortiz-Espín et al. 2017). Glutamate receptor homolog 3.4-mediated Ca^2+^ influx is involved in the regulation of seed germination under salt stress by modulating Na^+^ accumulation through the SOS (salt overly sensitive) pathway in Arabidopsis (Cheng et al. 2018). The Arabidopsis WD40 repeat-containing protein XIW1, which interacts with abscisic acid (ABA) insensitive 5 (ABI5) in the nucleus and maintains its stability, promotes salt inhibition of seed germination (Cai et al. 2020). The ABI4-RbohD (NADPH oxidase genes)/VTC2 (vitamin C defective 2) regulatory module promotes reactive oxygen species (ROS) accumulation to decrease seed germination under salinity stress (Luo et al. 2021). The Arabidopsis long hypocotyl 2 (HY2) gene acts as a positive regulator of NaCl signaling during seed germination (Piao et al. 2021). In rice, the main locus *qSE3*, which encodes the K^+^ transport gene *OsHAK21*, was reported to promote seed germination and seedling establishment under salt stress through ABA metabolism (He et al. 2019). The expression of the basic helix-loop-helix (bHLH) TF *OsbHLH035* is induced by salinity, and its mutants show delayed seed germination, particularly under salt stress conditions (Chen et al. 2018). The APETALA2 (AP2)-type transcription factor (TF), *SALT AND ABA RESPONSE ERF1* (*OsSAE1*), acts as a positive regulator of seed germination and salt tolerance in rice by repressing *OsABI5* expression (Li et al. 2022a). The exploration of the key genes associated with seed germination under salt stress deserves further investigation.

Genome-wide association studies (GWASs) are an effective strategy that use single-nucleotide polymorphisms (SNPs) as molecular genetic markers to detect valuable natural variation in trait-associated loci as well as allelic variations in candidate genes affecting complex traits (Huang et al. 2010; Zhao et al. 2011). With advances in gene sequencing and technical methods, GWASs have been successfully applied to determine quantitative trait loci (QTLs) and candidate genes of seed germination in plants, such as rice (Huang et al. 2021; Li et al. 2021a), maize (Huang et al. 2013), soybean (Kan et al. 2015; Liu et al. 2020), sorghum (Upadhyaya et al. 2016) and barley (Thabet et al. 2018). Recently, some studies involved in seed germination under salt stress have also been reported, such as in rice (Yu et al. 2018; Cui et al. 2018), barley (Mwando et al. 2020), flax (Li et al. 2022b), wheat (Hasseb et al. 2022) and oilseed rape (Zhang et al. 2022). However, it is unfortunate that the key genes of seed germination under salt stress identified via GWAS are still limited in rice.

In this study, we evaluated seed germination under salt stress using GR, GI, *T*_50_ and ML based on 168 diverse accessions from Rice Diversity Panel 1 (RDP1) (Zhao et al. 2011) in 2015 and 2017. The high-resolution, open-access research platform, which included 700 K SNP data (McCouch et al. 2016), was used for GWAS to identify the loci associated with rice seed germination under salt stress. Two candidate genes, the similar to transthyretin-like protein *OsTTL* and the serine/threonine protein kinase *OsSAPK1*, of a key locus *qNL3.1* were obtained. Our studies indicated that the *OsTTL* and *OsSAPK1* genes both positively regulated seed germination under salt stress, and their combination of excellent alleles had high potential for improving seed germination under salt stress in rice. It is helpful to elucidate the molecular mechanism of seed germination under salt stress and to improve seed germination in the direct seeding of rice, particularly in saline-alkali soil.

## Materials and methods

### Plant materials and growth

A total of 413 rice accessions from the Rice Diversity Panel 1 (RDP1) identified by Zhao et al. (2011) were obtained from Dr. Jian Hua at Cornell University (Table S1). Of these, 168 accessions, including 91 *JAPONICA* accessions, 56 *INDICA* accessions and 21 *ADMIX* accessions, were selected for evaluation of seed germination under salt stress. The mean germination rate (MGR) at 5 days after imbibition (DAI) of 168 accessions was more than 95% under H_2_O conditions in both 2015 and 2017. *Osttl* mutants (*Osttl-1*, *Osttl-2*, and *Osttl-3*) and *Ossapk1* mutants (*Ossapk1-1* and *Ossapk1-1*) were generated in the *japonica* Nipponbare background using the CRISPR/Cas9 system (Xing et al. 2014). All accessions were grown at the Jiangpu Experimental Station of Nanjing Agricultural University, Nanjing, Jiangsu. Field management was performed following the local standard methods (Cheng et al. 2015). All seeds were harvested at the maturity stage and dried at 42 ℃ for 7 days to break seed dormancy.

### Evaluation of seed germination

A total of 30 healthy grains of each accession were surface-sterilized with 0.5% sodium hypochlorite solution for 15 min and then rinsed three times with sterile distilled water. Seeds were imbibed in 9-cm Petri dishes with 40-mL quartz and 20 mL H_2_O solution in a growth chamber at 25 ± 1 ℃ with a 12 h/12 h light–dark cycle for 10 days*.* Salt treatment used 200 mM NaCl solution instead of H_2_O solution. Germination was considered visually by the emergence of the radicle through the hull by ≥ 2 mm, and seedling establishment was considered when the root length reached the seed length and the shoot length reached half of the seed length (Cheng et al. 2015). The germination rate (GR) was calculated after 10 days of imbibition. *T*_50_ was calculated using GERMINATOR software (Joosen et al. 2010). GI was calculated as GI = ∑ (Gt/*t*), where Gt is the number of germinated seeds on Day *t* (Wang et al. 2010). GR and GI were evenly divided into 10 levels from 1 to 10 and *T*_50_ from 10 to 1, respectively, according to Qiu et al. (2015). The germination mean level (ML) of each accession was calculated as $\frac{{{\text{ML\_GR}} + {\text{ML\_GI}} + {\text{ML}}\_T50}}{3}$ to evaluate the integrative capacity of seed germination under salt stress in this study. ML_GR, ML_GI and ML_*T*_50_ represent the mean levels of GR, GI and *T*_50_, respectively. Three replications of each accession were performed.

### Genome-wide association study

A high-density array of 700 K SNPs described by McCouch et al. (2016) was used in this study. SNPs with minor allele frequencies (MAFs) ≤ 5% and ≥ 25% missing ratios were filtered (Yano et al. 2016) using TASSEL 5.2.40 software (Bradbury et al. 2007). The final set of SNPs included 403,950 subsequently obtained for GWAS. Linear mixed-model association studies in *All* population, and two sub-populations, *INDICA* and *JAPONICA*, not in *ADMIX* group due to fewer accessions, were implemented using the Efficient Mixed-Model Association eXpedited (EMMAX) in Linux (Kang et al. 2010). The significance threshold for all indices was set to *P* ≤ 1.0e-5, indicated by a red horizontal line in the Manhattan plot at − log_10_*P* ≥ 5, according to Crowell et al. (2016). GWAS results of seed germination under salt stress were visualized in Manhattan and quantile‒quantile plots (Q-Q plots, Fig. S1) using the R package qqman. The clear peak signals with significant SNP clusters (at least three SNPs, any two significant SNPs within a 200 kb interval) were considered as one associated locus (Lv et al. 2016). The most significant (i.e., the highest − log_10_*P*) SNP in a cluster was considered to be the lead SNP. There was more noise in the Manhattan plots as *P* ≤ 1.0e-5 for six traits, including GR, *T*_50_ and ML in *INDICA* in 2015, GR and *T*_50_ in *INDICA* in 2017 and GR in *JAPONICA* in 2017. To reduce the risk of false-positive loci associated with six traits, a Bonferroni correction (Li et al. 2017) was applied. Those loci in which the *P* value thresholds of the lead SNP were higher than 1.24e-7 (0.05/403,950) were filtered. Finally, the identified loci, for which the physical interval of their lead SNPs was less than 100 kb, were integrated into a locus, named *qNLs*.

### Candidate gene analysis of *qNL3.1*

The candidate genomic region of the key locus *qNL3.1* was calculated according to ± 100 kb of the flanking lead SNPs, and a 301.042-kb genomic region was obtained refer to the intersection of two years. To identify the causal genes of the *qNL3.1* associated with seed germination under salt stress, the candidate genes were predicted with the Nipponbare reference genome (Lv et al. 2016) according to the MSU Rice Genome Annotation Project Release 7 database (http://rice.plantbiology.msu.edu). According to Yano et al. (2016), all the SNPs in the *qNL3.1* region were categorized into five groups as their functions using the RiceVarMap v2.0 database (http://ricevarmap.ncpgr.cn/). Group I included significant SNPs (− log_10_*P* ≥ 5) that caused amino acid exchange. Group II included significant SNPs that were located in the promoter region and 5′ noncoding sequence. Group III included significant SNPs that were located within a coding region but not predicted to change an amino acid, or an intron or a 3′ noncoding sequence. Group IV included significant SNPs that were located in the intergenic region. Group V included SNPs that were not significantly associated with seed germination.

### Expression analysis of candidate genes

The expression levels of five candidate genes, including *LOC_Os03g27320*, *LOC_Os03g27250*, *LOC_Os03g27280*, *LOC_Os03g27310* and *LOC_Os03g27360*, were predicted in shoots, roots and seedlings under salt stress by GENEVESTIGATOR. Seeds of Nipponbare were sampled at 0, 6, 12, 24, 36, 48, 60 and 72 h after imbibition under 200 mM NaCl and quickly frozen in liquid nitrogen. All samples were stored at -80 ℃ for RNA extraction.

Total RNA was isolated from approximately 80∼100 mg powder with a total RNA Kit (BioTeke, www.bioteke.com). The first-strand cDNA was synthesized with random oligonucleotides using the HiScript II reverse transcription kit (Vazyme Biotech, http://www.vazyme.com/) according to the manufacturer’s protocol. To measure the mRNA levels of genes, quantitative real-time PCR (RT‒qPCR) was conducted by a CFX96 Real-time System (BIO-RAD, USA) with SYBR Green Mix (Vazyme). The rice housekeeping gene *OsActin* (*LOC_Os03g50885*) was used as an internal control (Pei et al. 2020). The PCR conditions were as follows: 95 ℃ for 5 min; 36 cycles of 95 ℃ for 15 s, 58 ℃ for 30 s and 72 ℃ for 1 min; and 72 ℃ for 5 min. A final melt curve stage of 65–95 ℃ was performed to confirm the specificity of the primers. Relative quantification of the transcript levels was obtained based on the 2^−ΔΔ CT^ method (Livak and Schmittgen 2001). The amount of the transcripts in the dry Nipponbare seeds (0 h) was set at 1.0. All of the primer pairs had amplification efficiencies of approximately 100% and were designed according to http://quantprime.mpimp-golm.mpg.de/ and used for RT‒qPCR (Table S2). Three biological replicates were conducted.

### Generation and identification of transgenic plants

The CRISPR/Cas9 plasmid was designed according to a protocol described previously (Xing et al. 2014). Two target sites (target 1 and target 2) of *OsTTL* and *OsSAPK1* were confirmed via CRISPR-PLANT (http://www.genome.arizona.edu/crispr/CRISPRsearch.html), respectively. The target 964-bp segments that carry five elements, including target 1, gRNA-Sc, OsU3t, TaU3p and target 2, were amplified from the pCBC-MT1T2 vector using *OsTTL* (*OsSAPK1*)*-* BsF, F0, R0 and BsR primers. Then, the target 964-bp segments were cloned into the pHUE411 vector and verified using TaU3-FD2, TaU3-RD and OsU3-FD3 primers. The pCBC-MT1T2 and pHUE411 vectors were obtained from Qijun Chen’s laboratory (College of Biological Sciences, China Agricultural University, China). Transgenic rice plants were generated using the *Agrobacterium*-mediated cocultivation method in the background of Nipponbare. Genomic DNA was extracted from mutant seedlings using the cetyltrimethylammonium bromide (CTAB) method (Murray and Thompson 1980). The DNA fragments surrounding the two target sites of *OsTTL* and *OsSAPK1* were amplified as specific *OsTTL*-CRISPR-F/R and *OsSAPK1*-CRISPR-F/R primers, respectively, and directly sequenced to detect homozygous positive mutants. T_2_ plants of the CRISPR/Cas9 mutants were used for phenotype analysis. All the primers are listed in Table S2.

### Haplotype and combination analyses of *OsTTL *and *OsSAPK1*

For the *OsTTL* and *OsSAPK1* genes, the haplotypes were classified based on all SNPs with an MAF > 0.05 in a function range including the 5' flanking sequences of genes (≤ 2 kb from the first ATG) and the CDS of the target gene (Butardo et al. 2017). The haplotypes containing at least five investigated accessions were used for comparative analysis (Wang et al. 2015). According to the haplotypes of *OsTTL* and *OsSAPK1*, the phenotypes of all accessions combined were compared for excellent combinations of *OsTTL* and *OsSAPK1* to improve seed germination under salt stress.

### Identification of salt-tolerant accessions

The phenotypic values of the ML showed the integrative capacity of seed germination under salt stress. The 5% of accessions with the highest phenotypic values of seed germination under salt stress were selected based on the ML. The alleles of the causal genes *OsTTL* and *OsSAPK1* of *qNL3.1* were analyzed among the selected accessions, and the best cross combinations were predicted to improve rice seed germination under salt stress.

### Data analysis

Phenotypic data and variance were calculated by Excel 2017 software. The significant differences were tested using Student’s *t* test or Fisher’s least significant difference (LSD) test at the 5% and 1% levels of probability. Broad-sense heritability among 168 accessions was calculated using the method described by Lu et al. (2015). The correlation coefficients for multiple indices related to seed germination were calculated using the R package corrplot.

## Results

### Characterization of seed germination under salt stress

The 168 accessions of rice were evaluated via four indices of seed germination, including GR, GI, *T*_50_ and ML, under salt stress in 2015 and 2017 (Table S1). Phenotypic statistics showed that the GR, GI, *T*_50_ and ML of this population under salt stress ranged from 24.00 to 100.00%, 1.04 to 13.13, 1.89 to 12.00, and 1.67 to 9.67, respectively. The average values of the GR, GI, *T*_50_ and ML in 2015 and 2017 were 93.76% and 93.84%, 7.54 and 6.96%, 3.62 and 4.20%, and 7.72 and 7.35%, respectively (Table 1). Approximately normal distributions were observed in GI, *T*_50_ and ML, and skewed distributions were observed in GR in the two years (Fig. S2a–d), revealing that there was extensive phenotypic variation in GR, GI, *T*_50_ and ML under salt stress among this population.

**Table 1:** Descriptive statistics of GR, *T*_50_, GI and ML under salt stress in 2015 and 2017

Sub-populations	Traits	Years	Max	Min	Mean	SD	CV	Mean CV	Heritability (%)	*G *× E
*All*	GR	2015	100.00	36.67	93.76	8.40	0.09	0.10	54.55	***
		2017	100.00	24.00	93.84	10.20	0.11			
	GI	2015	13.13	1.67	7.54	1.72	0.23	0.25	72.07	***
		2017	11.80	1.04	6.96	1.89	0.27			
	*T*_50_	2015	12.00	1.89	3.62	1.27	0.35	0.35	77.09	***
		2017	12.00	2.06	4.20	1.45	0.35			
	ML	2015	9.67	2.00	7.72	0.97	0.13	0.14	71.50	***
		2017	9.00	1.67	7.35	1.10	0.15			
*INDICA*	GR	2015	100.00	36.67	93.24	11.07	0.12	0.14	82.27	***
		2017	100.00	24.00	92.67	14.14	0.15			
	GI	2015	10.63	1.67	7.57	1.85	0.24	0.28	81.63	***
		2017	11.80	1.04	7.32	2.36	0.32			
	*T*_50_	2015	12.00	2.32	3.71	1.74	0.47	0.48	87.11	***
		2017	12.00	2.06	4.15	2.01	0.48			
	ML	2015	9.00	2.00	7.70	1.21	0.16	0.18	84.36	***
		2017	9.00	1.67	7.39	1.48	0.20			
*JAPONICA*	GR	2015	100.00	63.33	94.01	7.07	0.08	0.08	12.62	***
		2017	100.00	64.61	94.55	7.63	0.08			
	GI	2015	10.02	3.56	7.54	1.53	0.20	0.20	58.07	***
		2017	9.13	3.26	6.77	1.39	0.20			
	*T*_50_	2015	6.95	2.35	3.54	0.92	0.26	0.24^c^	55.15	***
		2017	7.01	2.90	4.22	0.97	0.23			
	ML	2015	8.67	5.00	7.74	0.81	0.10	0.11	48.71	***
		2017	8.33	4.67	7.33	0.79	0.11			

***Indicates *P* < 0.001, *G *× *E* indicates interaction of genotype and environment

By comparison among the *All*, *JAPONICA* and *INDICA* subgroups, there were no significant differences in the mean values in GR, GI, *T*_50_ and ML during seed germination under salt stress (Table S1; Fig. S2 e–h). However, the CVs of GR, GI, *T*_50_ and ML in the *INDICA* subgroup were higher than those in the *JAPONICA* subgroup (Table 1), suggesting that the *INDICA* subgroup may have a relatively larger phenotypic variation than the *JAPONICA* subgroup. In addition, two-way analysis of variance (ANOVA) revealed that the broad-sense heritabilities of GR, GI, *T*_50_ and ML were more than 65% in 2015 and 2017, and the G × E interactions were all significant (*P* < 0.001) (Table 1). These results suggested that seed germination under salt stress is regulated by both genetic and environmental factors.

**Fig. 1:**
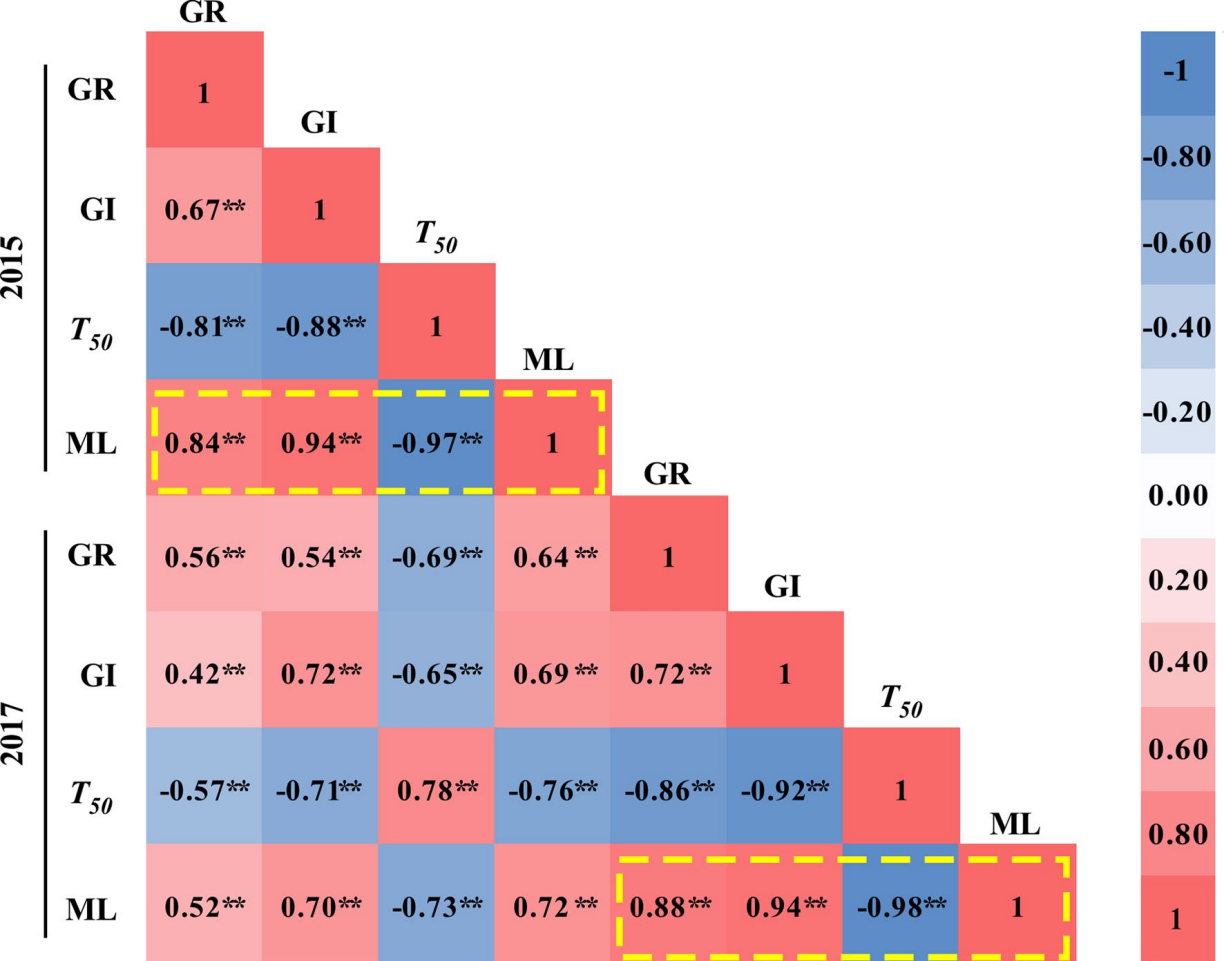
Heatmap depicting the correlations among GR, GI, *T*_50_ and ML under salt stress in 2015 and 2017. **Indicates significant correlations using a two-tailed *t* test (*P* < 0.01). Red and blue indicate positive and negative correlations, respectively. The numerical value is Pearson’s correlation coefficient

Furthermore, we calculated the correlation coefficients among the four indices of seed germination under salt stress over two years. There were significantly positive correlations among GR, GI and ML and negative correlations between *T*_50_ and the other three indices (Fig. 1). The highest correlation coefficients were found between ML and the other three indices, suggesting that ML might be a more effective index for the evaluation of seed germination under salt stress.

### Loci associated with seed germination under salt stress

**Fig. 2:**
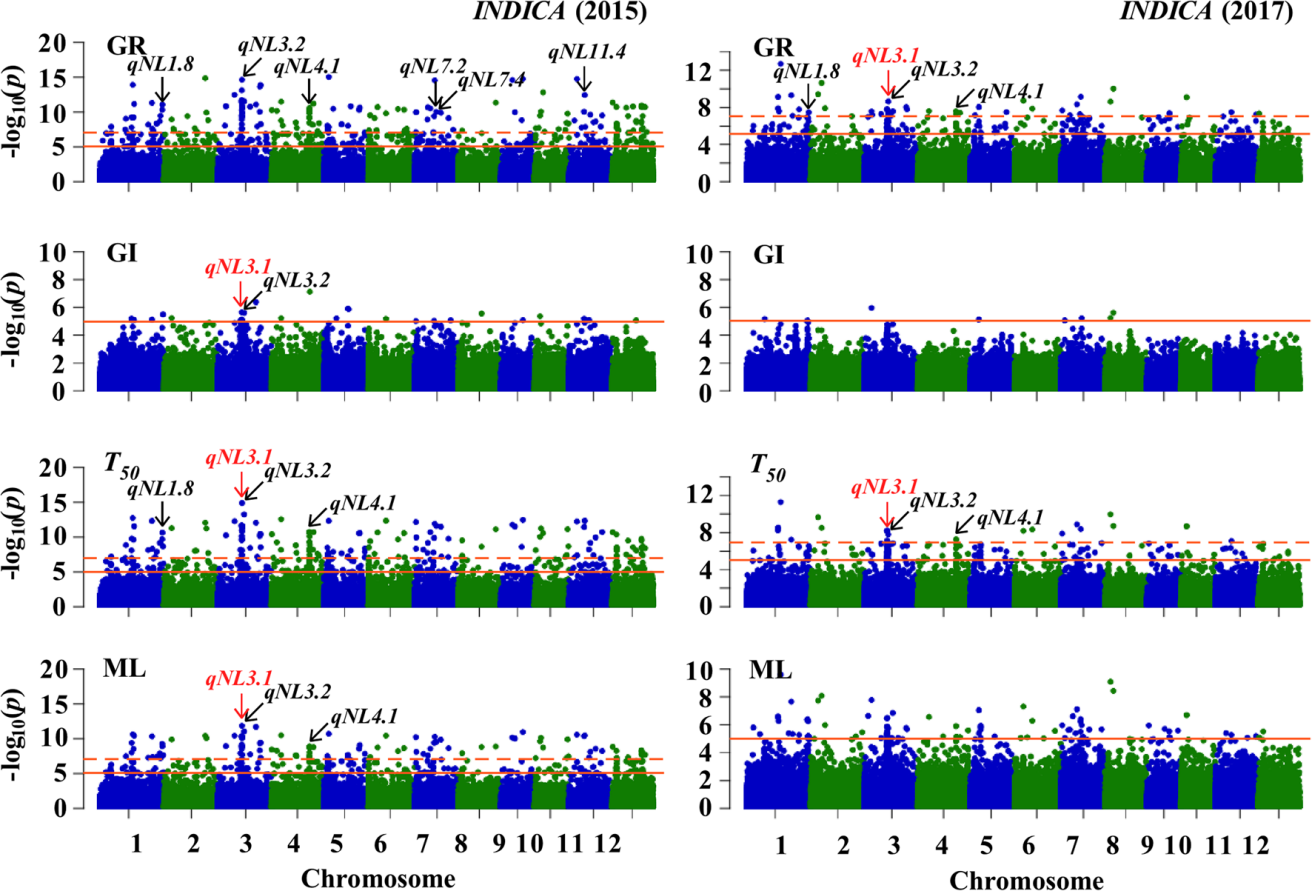
Genome-wide association analysis of GR, GI, *T*_50_ and ML in *INDICA*. Manhattan plots of GR, GI, *T*_50_ and ML in 2015 (left) and 2017 (right). The horizontal red solid lines indicate a statistically significant threshold of *P* < 1 × 10^–5^. The horizontal red dotted lines indicate the Bonferroni-corrected threshold of *P* < 1 × 10^–7^. The arrows indicate the loci identified in both years (colour figure online)

**Fig. 3:**
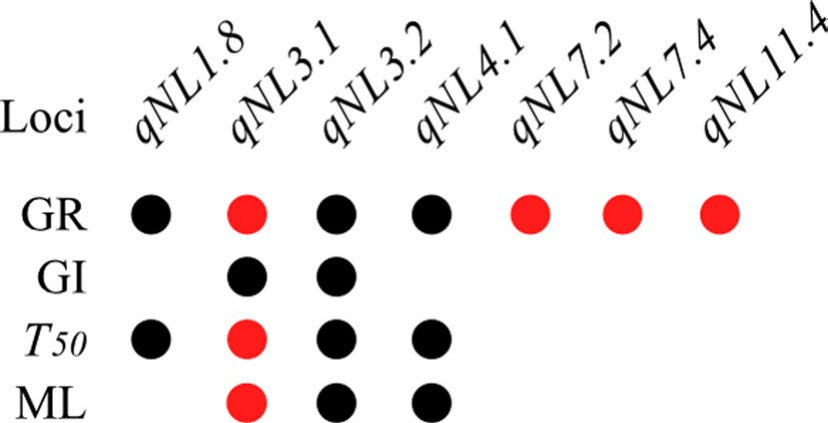
Analysis of seven loci identified in both years based on the GR, GI, *T*_50_ and ML via GWAS. The black circle indicates the loci identified in the *INDICA* group; the red circle indicates the loci identified in the *All* and *INDICA* groups (colour figure online)

Based on four indices, GR, GI, *T*_50_ and ML, in the two years, we conducted GWAS for seed germination under salt stress using the Efficient Mixed-Model Association eXpedited (EMMAX) model according to Crowell et al. (2016). A total of 56 loci were significantly associated with the four indices for seed germination under salt stress, including 35 in 2015 and 21 in 2017 (Figs. 2 and S3–4; Table S3). Seven loci, including *qNL1.8*, *qNL3.1*, *qNL3.2*, *qNL4.1*, *qNL7.2*, *qNL7.4* and *qNL11.4*, were simultaneously identified in 2015 and 2017 (Figs. 2 and 3); as a result, 49 loci in total were identified in two years in this study. Compared with the QTLs reported, 16 loci were colocalized with the reported QTLs, and the remaining 33 loci might be the novel loci identified in this study (Table S3).

Among the seven loci simultaneously identified in the two years, two loci*, qNL3.1* and *qNL3.2*, were associated with all four indices of seed germination under salt stress, and three loci, *qNL3.1*, *qNL3.2* and *qNL4.1*, were associated with at least three indices of seed germination (Fig. 3). In addition, seven loci, *qNL1.10*, *qNL3.1*, *qNL5.4, qNL7.2, qNL7.4*, *qNL11.2* and *qNL11.4*, were identified in both the *All* and *INDICA* groups; two loci, *qNL8.2* and *qNL7.6*, were identified in both the *All* and *JAPONICA* groups, and no colocalized loci were identified in both the *JAPONICA* and *INDICA* groups (Table S3).

One locus, *qNL3.1*, identified in both the *All* and *INDICA* groups was associated with the four indices in the two years (Figs. 2 and 3), indicating that it might be a key locus for rice seed germination under salt stress. We found that the key *qNL3.1* was colocalized with *qLTG-3* (Jiang et al. 2006), and its region contained the serine/threonine protein kinase *OsSAPK1* (*LOC_Os03g27280*). Mutants of the *OsSAPK1* gene were reported to exhibit lower germination rates under salt stress than wild-type plants (Lou et al. 2018). Therefore, we focused on *qNL3.1* for further investigation.

### Candidate genes of the key *qNL3.1*

**Fig. 4:**
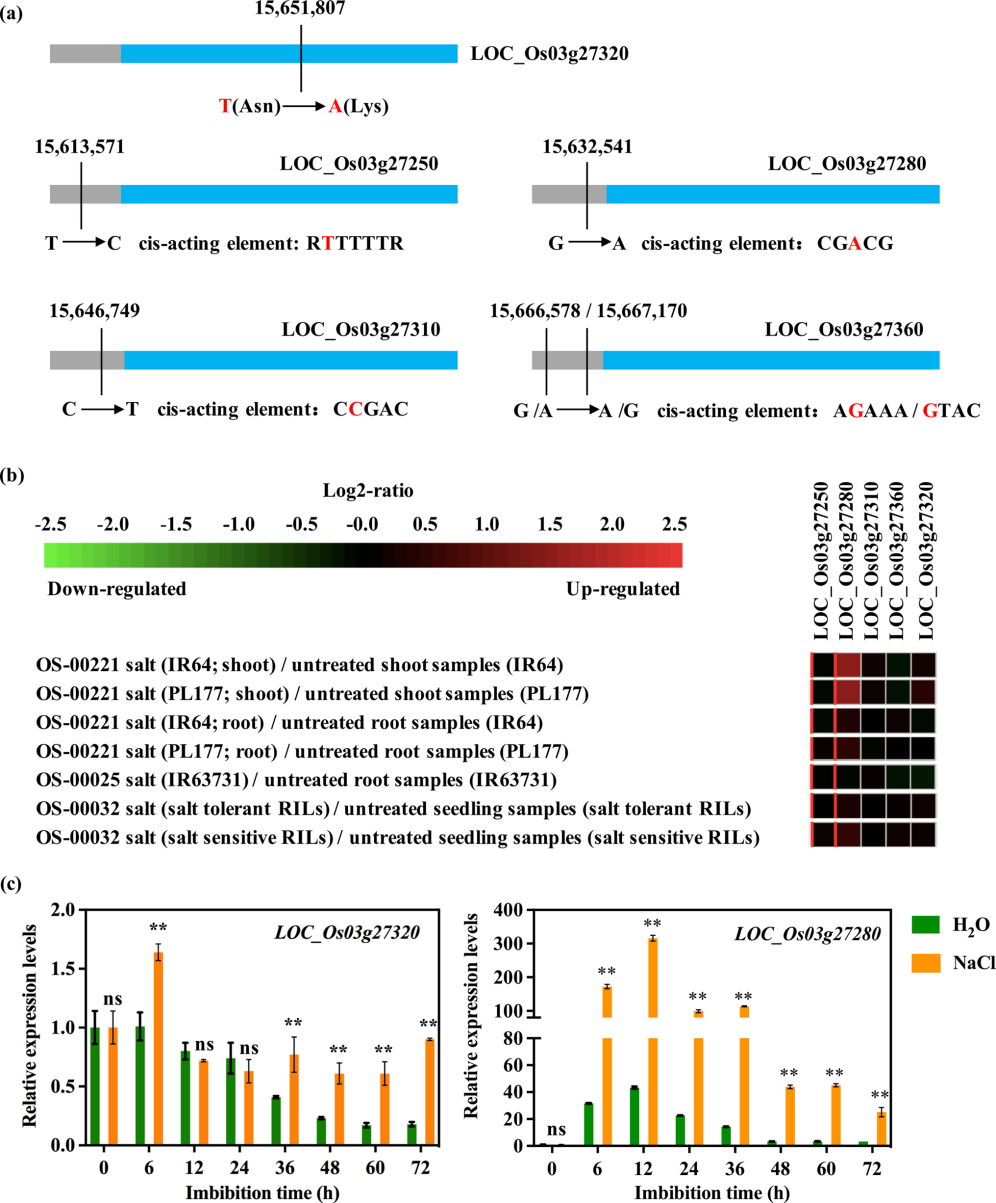
Analysis and expression levels of candidate genes for the key locus *qNL3.1*. **a** Information on significant SNPs in candidate genes. The gray and blue bars indicate the promoter regions and CDSs of candidate genes, respectively. The red font indicates a variant base or amino acid. **b** Heatmap showing the differential expression of candidate genes of *qNL3.1* based on the array database deposited in GENEVESTIGATOR. Red, upregulation; green, downregulation. **c** Expression patterns of *OsTTL* (*LOC_Os03g27320*) and *OsSAPK1* (*LOC_Os03g27280*) under H_2_O and NaCl conditions. The relative expression levels were calibrated against the expression of the *OsActin* gene and quantified by RT‒qPCR. **Indicates a significant difference at the 0.01 level. ns indicates not statistically significant (colour figure online)

To identify the causal gene of *qNL3.1* controlling seed germination under salt stress, we analyzed all the candidate genes within a 301.042 kb region of *qNL3.1* with the Nipponbare reference genome (http://rice.plantbiology.msu.edu) and identified 51 candidate genes after the removal of 16 genes encoding transposons and retrotransposons (Table S4). The classified results according to Yano et al. (2016) showed that only five genes were targeted and associated with the significant SNPs of *qNL3.1* (Table S5)*. LOC_Os03g27320*, located within Group I, had one significant SNP, SNP-3.15650453, which resulted in nonsynonymous variants on the CDS with amino acid residue changes from lysine (Lys) in accessions with “AA” to asparagine (Asn) in accessions with “TT” (Lys309Asn) (Fig. 4a; Table S5), indicating that it might be one of the causal genes of *qNL3.1*. The other four genes, *LOC_Os03g27250*, *OsSAPK1* (*LOC_Os03g27280*), *LOC_Os03g27310* and *LOC_Os03g27360*, were located within Group II. The significant SNPs SNP-3.15612217, SNP-3.15631187 and SNP-3.15645395 were located in the promoter regions of *LOC_Os03g27250*, *OsSAPK1* and *LOC_Os03g27310,*, respectively, and SNP-3.15665224 and SNP-3.15665816 were located in the promoter regions of *LOC_Os03g27360* (Fig. 4a; Table S5). The five SNPs were located on the *cis*-acting elements CGACC, RTTTTTR, CCGAC, AGAAA and GTAC. These results suggest that the expression levels of the four genes may be tightly associated with seed germination under salt stress.

*LOC_Os03g27320*, *LOC_Os03g27250*, *OsSAPK1*, *LOC_Os03g27310* and *LOC_Os03g27360* encode a steroid-binding protein, OsFBO14, F-box and another domain containing protein, CAMK_CAMK_like.19. CAMK includes calcium/calmodulin dependent protein kinases*,* histone H3, and the RING-H2 finger protein ATL5H precursor (Table S5), respectively.

The expression profiles of the five candidate genes of *qNL3.1* in shoots, roots and seedlings under salt stress were determined based on the array database deposited in GENEVESTIGATOR. The results showed that the expression levels of only *OsSAPK1* in the shoots and seedlings were significantly upregulated under salt stress (Fig. 4b). Furthermore, we detected the expression of *LOC_Os03g27320* and *OsSAPK1* with the RT‒qPCR approach during seed germination under H_2_O and 200 mM NaCl conditions. The expression of *LOC_Os03g27320* gradually decreased from 6 to 72 h after imbibition under H_2_O conditions, and the expression levels of *OsSAPK1* increased from 0 to 12 h after imbibition and then decreased (Fig. 4c). Compared with that under H_2_O conditions, the expression levels of *LOC_Os03g27320* were upregulated after imbibition for 6 h and from 36 to 72 h under 200 mM NaCl conditions, and the expression level of *OsSAPK1* was dramatically upregulated during seed germination (Fig. 4c). Overall, *LOC_Os03g27320* and *OsSAPK1* were both induced by salt treatment and might be causal genes of *qNL3.1*.

### Effect of seed germination of *Osttl* mutants under salt stress

**Fig. 5:**
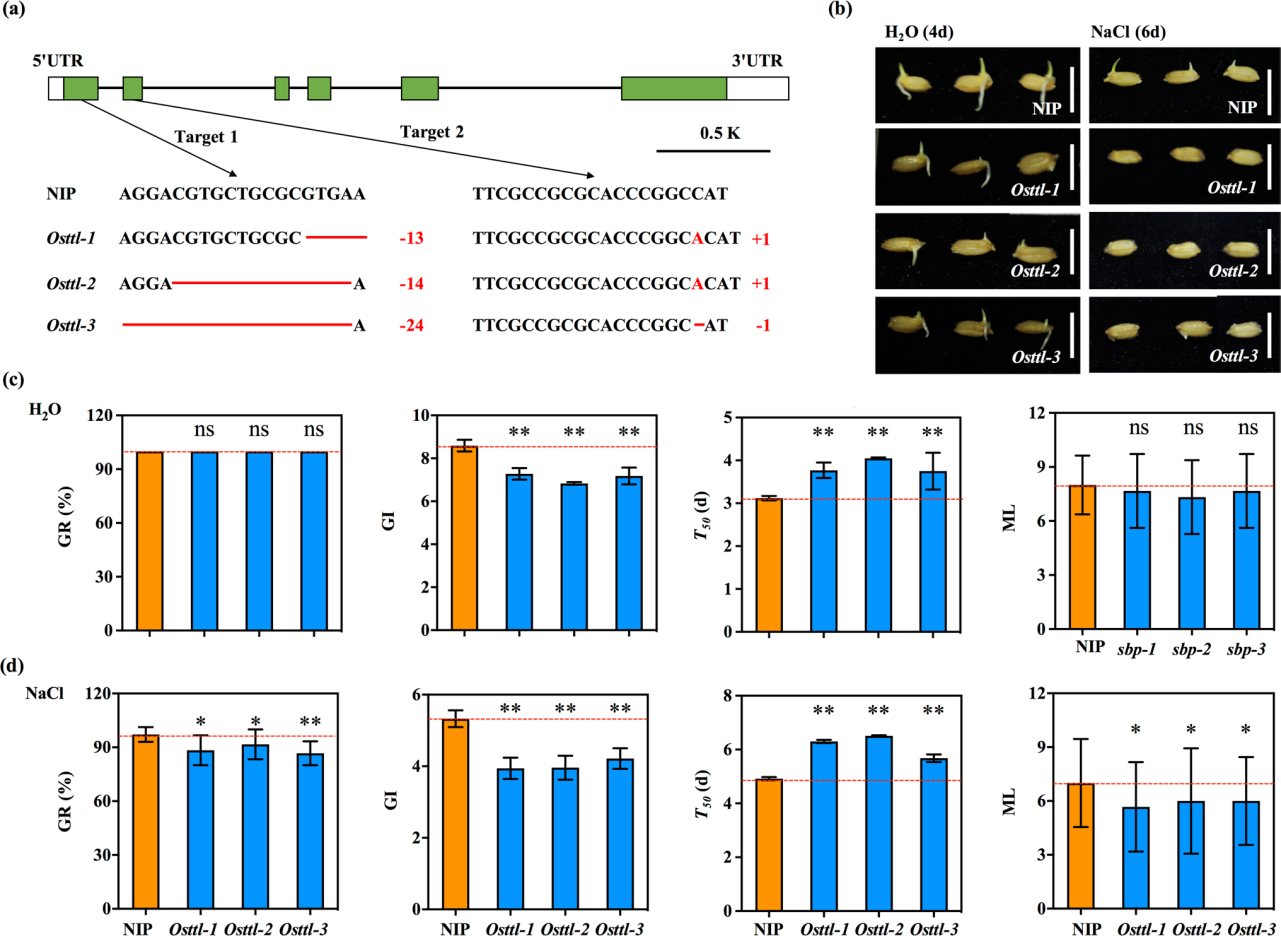
Seed germination is regulated by *OsTTL* under H_2_O and salt stress*.* **a** Generation of *Osttl* mutants via the CRISPR/Cas9 system. Green rectangles represent the exons of *OsTTL*. White rectangles represent the UTRs of *OsTTL*. Red lines and bases represent deletions or insertions in the genome of *OsTTL.* **b** Seed germination of *Osttl* mutants and WT after 4 and 6 days of imbibition under H_2_O and NaCl conditions. Comparison of GR, GI, *T*_50_ and ML between the *Osttl* mutants and WT under H_2_O (**c**) and NaCl (**d**) conditions. Bars = 5 cm. The red horizontal dotted lines indicate the mean value of WT. *, ** Indicates significant differences at the 0.05 and 0.01 levels, respectively. ns indicates not statistically significant (colour figure online)

*LOC_Os03g27320* is described similar to the transthyretin-like (TTL) protein with 334 amino acids (Fig. S5), designated *OsTTL*. To characterize the function of *OsTTL* in seed germination under salt stress, we employed the CRISPR/Cas9 system to generate mutants of *OsTTL* with *japonica* Nipponbare and obtained three *Osttl* mutants, *Osttl-1*, *Osttl-2* and *Osttl-3* (Fig. 5a). *Osttl-1* contained a 13-bp (GTGAACGGGAGCC) deletion in the first exon and an “A” insertion in the second exon of *OsTTL*; *Osttl-2* contained a 14-bp (CGTGCTGCGCGTGA) deletion in the first exon and an “A” insertion in the second exon of *OsTTL*; and *Osttl-3* contained a 24-bp (CCCGTCGAGGACGTGCTGCGCGTG) deletion in the first exon and an “A” deletion in the second exon of *OsTTL* (Fig. 5a). As predicted based on these nucleotide sequences, the amino acid sequence of OsTTL in *Osttl-1* contains 330 amino acids, breaking the 2-oxo-4-hydroxy-4-carboxy-5-ureidoimidazoline decarboxylase (OHCU_decarbox) domain; the amino acid sequences of OsTTL in *Osttl-2* and *Osttl-3* contain 106 and 102 amino acids, respectively, due to the premature termination of *OsTTL* (Fig. S5). These results indicated that the three *Osttl* mutants might lack the function of *OsTTL*.

The progeny of three homozygous *Osttl* mutants (T_2_) was selected, and their GR, GI, *T*_50_ and ML were evaluated during seed germination under H_2_O and 200 mM NaCl conditions. Under H_2_O conditions, the GR and ML values in *Osttl-1*, *Osttl-2* and *Osttl-3* were not significantly different from those in WT, but the GI significantly decreased and the *T*_50_ significantly increased (Fig. 5b–c). Under salt conditions, the GR, GI and ML values in *Osttl-1*, *Osttl-2* and *Osttl-3* were significantly decreased compared to those in WT, and the *T*_50_ significantly increased (Fig. 5b, d). In addition, the seedling rate (SR) was measured during seed germination under both H_2_O and 200 mM NaCl conditions. The results showed that the SR values in *Osttl-1*, *Osttl-2* and *Osttl-3* were significantly decreased compared to that in WT under both H_2_O and 200 mM NaCl conditions (Fig. S6). These results demonstrated that the *Osttl* mutants resulted in low germination speed and seedling growth during seed germination under salt stress, suggesting that *OsTTL* could positively regulate seed germination under salt stress.

### Effect of seed germination of *Ossapk1* mutants under salt stress

**Fig. 6:**
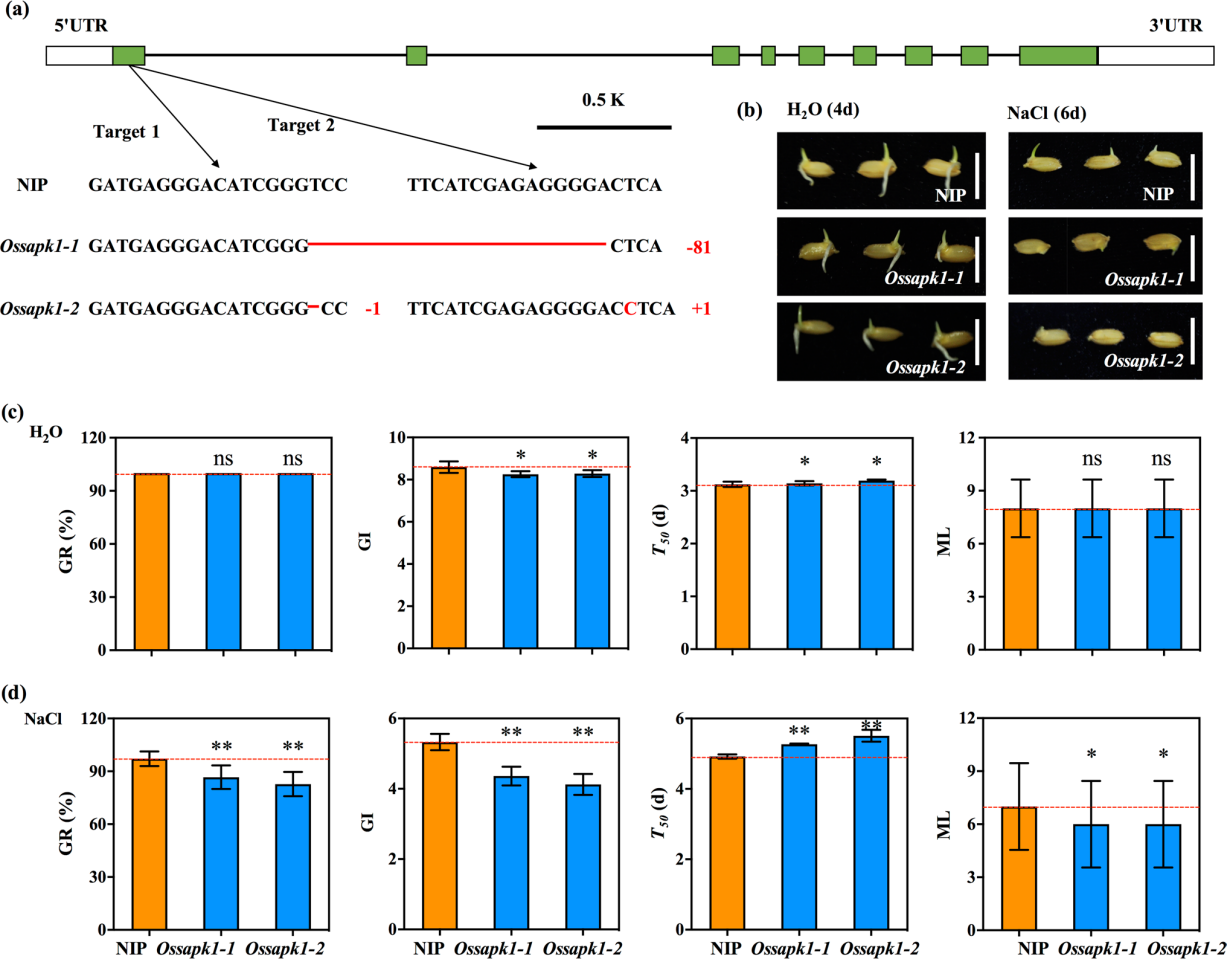
Seed germination is regulated by *OsSAPK1* under H_2_O and salt stress. **a** Generation of *Ossapk1* mutants via the CRISPR/Cas9 system. Green rectangles represent the exons of *OsSAPK1*. White rectangles represent the UTRs of *OsSAPK1*. Red lines and bases represent deletions or insertions in the genome of *OsSAPK1.* **b** Seed germination of *Ossapk1* mutants and WT after 4 and 6 days of imbibition under H_2_O and NaCl conditions. Comparison of GR, GI, *T*_50_ and ML between the *Ossapk1* mutants and WT under H_2_O (**c**) and NaCl (**d**) conditions. Bars = 5 cm. The red horizontal dotted lines indicate the mean value of WT. *, ** indicate significant differences at the 0.05 and 0.01 levels, respectively. ns indicates not statistically significant (colour figure online)

To confirm the function of *OsSAPK1* in rice seed germination under salt stress, we employed the CRISPR/Cas9 system to generate mutants of *OsSAPK1* with *japonica* Nipponbare and obtained two mutants, *Ossapk1-1* and *Ossapk1-2* (Fig. 6a). *Ossapk1-1* contained an 81-bp (TCCGGGAACTTCGGGGTGGCCAAGCTCGTCCGCGACGTCGCCACCAACCACCTCTTCGCCGTCAAGTTCATCGAGAGGGGA) deletion in the first exon of *OsSAPK1*, and *Ossapk1-2* contained a “T” deletion and a “C” insertion in the first exon of *OsSAPK1* (Fig. 6a). The product of *OsSAPK1* has 342 amino acids. As predicted based on these nucleotide sequences, the amino acid sequences of OsSAPK1 in *Ossapk1-1* and *Ossapk1-2* contained 315 and 342 amino acids, breaking their serine/threonine protein kinase domains (Fig. S7). These results indicated that the two *Ossapk1* mutants might lack the function of *OsSAPK1*.

The progeny of two homozygous *Ossapk1* mutants (T_2_) was selected, and their GR, GI, *T*_50_ and ML values were evaluated during seed germination under H_2_O and 200 mM NaCl conditions. Under H_2_O conditions, the GR and ML in *Ossapk1-1* and *Ossapk1-2* were not significantly different from those in WT, but the GI significantly decreased, and the *T*_50_ significantly increased (Fig. 6b–c). Under salt conditions, the GR, GI and ML in *Ossapk1-1* and *Ossapk1-2* were significantly decreased compared to those in WT, and the *T*_50_ significantly increased (Fig. 6b, d). Similarly, the SR was measured during seed germination under H_2_O and 200 mM NaCl conditions. The results showed that the SR in *Ossapk1-1* and *Ossapk1-2* were significantly decreased compared to that in WT under both H_2_O and 200 mM NaCl conditions (Fig. S8). These results demonstrated that the *Ossapk1* mutants resulted in low seed germination and seedling growth during seed germination under both H_2_O and 200 mM NaCl conditions, suggesting that *OsSAPK1* could positively regulate rice seed germination under salt stress.

### Haplotypes and their combinations of *OsTTL *and *OsSAPK1*

**Fig. 7:**
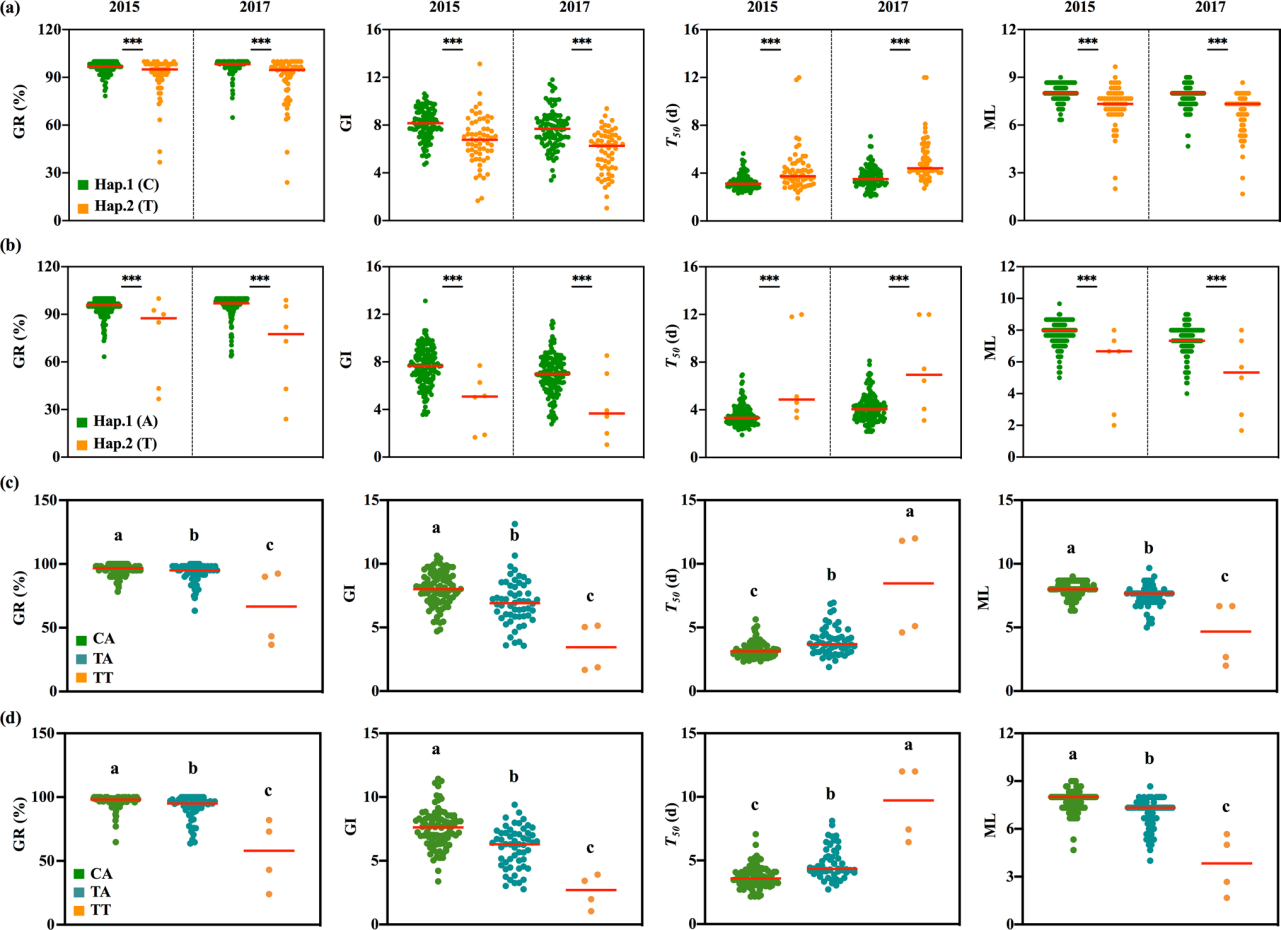
Plots of GR, GI, *T*_50_ and ML of accessions containing different haplotypes in 2015 and 2017. **a** Plots of GR, GI, *T*_50_ and ML of accessions containing the Hap.1 (C) and Hap.2 (T) haplotypes of *OsTTL*. **b** Plots of GR, GI, *T*_50_ and ML of accessions containing the Hap.1 (A) and Hap.2 (T) haplotypes of *OsSAPK1*. Haplotype combinations of *OsTTL* and *OsSAPK1* in 2015 (**c**) and 2017 (**d**). ***Indicates a significant difference at the 0.001 level. Different lowercase letters indicate significant differences at the 0.05 level

Based on significant SNPs located on these two causal genes, 2 and 2 haplotypes were detected in the *OsTTL* and *OsSAPK1* genes among the 168 accessions, respectively (Fig. 7a–b). For the *OsTTL* gene, the GR and ML of Hap.1 (C) were significantly higher than those of Hap.2 (T) under salt stress in both 2015 and 2017, and the *T*_50_ was significantly lower during seed germination (Fig. 7a). For the *OsSAPK1* gene, the GR and ML of Hap.1 (A) were significantly higher than those of Hap.2 (T) under salt stress in both 2015 and 2017 (Fig. 7b), and the *T*_50_ was significantly lower during seed germination (Fig. 7b). These results suggest that both Hap.1 of *OsTTL* and Hap.1 of *OsSAPK1* could result in higher seed germination under salt stress.

Meanwhile, the combination analysis of the *OsTTL* and *OsSAPK1* haplotypes showed that the combination (CA) of the excellent Hap.1 (C) of *OsTTL* and Hap.1 (A) of *OsSAPK1* was significantly higher than other haplotypic combinations (TA and TT) in terms of GR, GI and ML in both 2015 and 2017 (Fig. 7c–d) and significantly lower than that in *T*_50_ (Fig. 6c–d), suggesting that the combination of the excellent alleles of *OsTTL* and *OsSAPK1* had the potential to improve seed germination under salt stress in rice.

### Identification of salt-tolerant accessions during seed germination

**Fig. 8:**
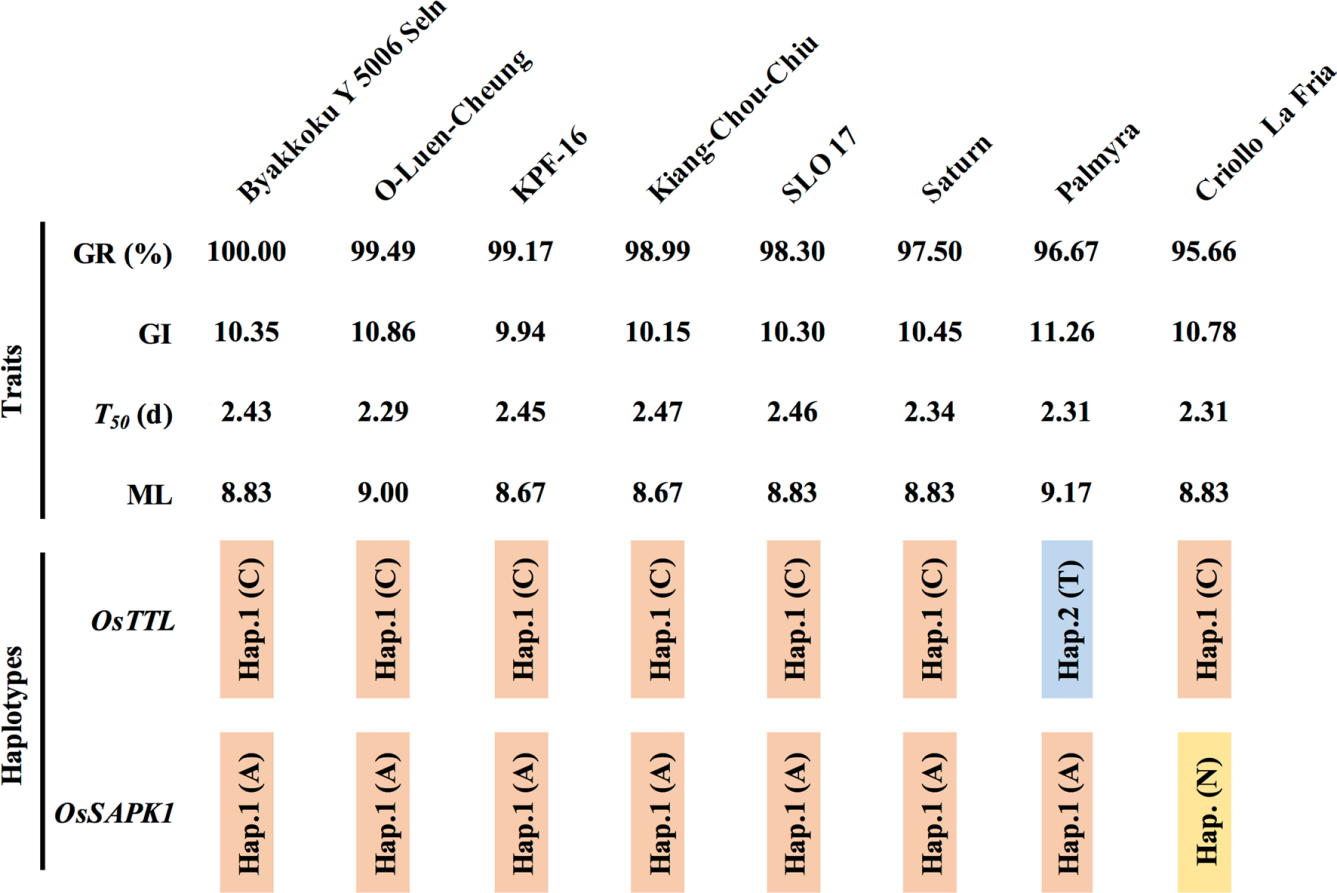
Eight elite accessions were selected from RDP1. GR, GI, *T*_50_ and ML (up) values and the haplotypes (down) of *OsTTL* and *OsSAPK1* of elite accessions

Given the phenotype of seed germination of accessions under salt stress, the top eight elite accessions were identified from 168 accessions, including 5 accessions in the *IND* group, O-Luen-Cheung, Byakkoku Y 5006 Seln, Criollo La Fria, SLO 17 and Kiang-Chou-Chiu, and 3 accessions in the *ADMIX* group, Palmyra, Saturn and KPF-16 (Fig. 8). Interestingly, the six elite accessions all carried the excellent Hap.1 of *OsTTL* and Hap.1 of *OsSAPK1* (Fig. 8), indicating that these accessions could be selected with improved seed germination under salt stress in rice breeding.

## Discussion

Seed germination is a critical stage initiating the life cycle of a plant. In this study, to evaluate seed germination under salt stress, we selected 200 accessions with adjacent heading dates (growth duration) grown in Nanjing from 413 accessions (RDP1, Zhao et al. 2011). To further decrease the environmental effect, we deleted another 32 accessions with abnormal germination rates under normal conditions in this study, and 168 were subjected to the experiment for two years. These accessions had extensive phenotypic variation in seed germination under salt stress in GR, GI, *T*_50_ and ML, suggesting that they might be suitable for a GWAS. Moreover, *INDICA* exhibited larger phenotypic variation than *JAPONICA*, consistent with the results reported by Yang et al. (2022), indicating that there are likely some key genes regulating seed germination under salt stress in *INDICA*. Previous studies showed that *INDICA* accessions presented stronger seed vigor during seed germination than *JAPONICA* (Wang et al. 2010), while *JAPONICA* was more tolerant to cold and salt during seed germination than *INDICA* (Pan et al. 2015; Islam et al. 2022). No significant differences were observed in seed germination under salt stress between *INDICA* and *JAPONICA* in our study. This is different from the results under salt stress reported by Islam et al. (2022), possibly due to the differences in their populations and salt concentrations.

The identification of QTLs controlling seed germination under salt stress would contribute to understanding the genetic control and elucidating the processes of seed germination under salt stress. In this study, we identified 49 loci significantly associated with seed germination under salt stress via GWAS in 2015 and 2017 and found that seven loci, *qNL1.8*, *qNL3.1*, *qNL3.2*, *qNL4.1*, *qNL7.2*, *qNL7.4* and *qNL11.4*, were identified in both years, suggesting that their genetic effects were relatively stable in rice fields. These stable loci are worthy of further exploration for the novel genes regulating seed germination under salt stress, which would aid in understanding the genetic control and elucidating the processes of seed germination under salt stress in rice. The other 42 loci were not identified in both years, which may be due to different states of grain development because of the environmental conditions. Comparing chromosomal locations of reported QTLs, there were approximately one-third of loci colocated with the reported QTLs. For example, *qNL6.3* was close to the position of *qRI6* (Islam et al. 2022), including the *OsSAE1* gene (Li et al. 2022b), which regulates seed germination under salt stress. *qNL1.1* and *qNL1.6* were colocated with the positions of *OsNLP3* (Yi et al. 2022) and *OsMYB3R-2* (Dai et al. 2007) regulating seed germination under salt stress, respectively. *qNL11.3* was colocated with the position of *Rab16A* (Ganguly et al. 2012), enhancing salt tolerance at the seedling stage. This finding suggests that the identified loci of seed germination under salt stress in our study with GR, GI, *T*_50_ and ML by GWAS are reliable.

It is well known that the selection of effective indices is important for evaluating targeted traits correctly. In previous studies, Wang et al. (2010) evaluated the phenotype of seed germination under H_2_O and salt conditions using the imbibition rate and germination percentage and identified 16 QTLs of rice seed germination via the recombinant inbred lines (RILs, F_2:9_) population derived from a cross between *japonica* Jiucaiqing and *indica* IR26. Zeng et al. (2021) also assessed the phenotype of seed germination under H_2_O and salt conditions using GR and GI and identified 13 QTLs for seed germination via the BC_1_F_2_ population derived from the crossing Wujiaozhan/Nip (Nipponbare)//Nip. Lai et al. (2016) determined the characteristics of seed vigor using the germination potential, GR, GI and *T*_50_ and identified 19 additive and 2 epistatic quantitative trait loci for seed vigor via RIL populations. Shi et al. (2017) analyzed salt tolerance at the seed germination stage using the stress susceptibility indices of vigor index and mean germination time based on 478 rice accessions and identified eleven significantly associated loci via GWAS. This shows that seed germination is a complicated trait that can be evaluated using several indices, e.g., GR, GI and *T*_50_. However, there are few comprehensively evaluated indices used. In our study, we divided GR, GI and *T*_50_ into 10 levels according to Qiu et al. (2015) and set the ML index through the integrated GR, GI and *T*_50_ indices. Our results showed that 42, 4, 27 and 23 loci were significantly associated with GR, GI, *T*_50_ and ML, respectively, suggesting that the number of identified loci with ML is relatively appropriate. Meanwhile, of 23 loci identified by ML, 18, 4 and 18 loci colocalized with GR, GI and *T*_50_, respectively, and significant and tight correlations of ML with the GR, GI and *T*_50_ indices were observed, implying that ML, as a comprehensive index, is effective for the evaluation of seed germination.

In *Arabidopsis thaliana*, the protein TTL is a potential substrate of BR-INSENSITIVE-1 (BRI1) and is involved in brassinosteroid (BR)-mediated plant growth (Nam et al. 2004). BRs, as a class of polyhydroxylated steroid plant hormones, participate in the regulation of seed germination (Steber and McCourt 2001; Bajguz et al. 2020) as well as in responding to environmental stresses (Soares et al. 2020; Kim et al. 2019). In rice, Xiong et al. (2022) reported that BRs could regulate rice seed germination through the BZR1 (brassinazole-resistant 1)-*RAmy3D* (alpha-amylase 3D) transcriptional module. TTL acts as a bifunctional enzyme, 5-hydroxyisocyanate (5-HIU) hydrolase and 2-oxo-4-hydroxy-4-carboxy-5-ureidoimidazoline decarboxylase (OHCU_decarbox), which catalyze two steps in the allantoin biosynthesis pathway (Lamberto et al. 2010; Pessoa et al. 2010). In addition, Liu et al. (2020) reported that allantoin might be a key regulator of sugar beet salt tolerance. As predicted by https://www.uniprot.org/, the OsTTL protein contained OHCU_decarbox and transthyretin (TR_THY, Fig. S5), and seed germination and seedling growth in *Osttl* mutants were significantly reduced under salt stress. These findings suggest that *OsTTL* plays important roles in seed germination under salt stress in rice and that the salt tolerance mechanism may be involved in the BR and allantoin biosynthesis pathways.

It is well known that SnRK2s (sucrose nonfermenting1-related protein Kinase 2), as core components of the ABA signaling pathway, bind to and phosphorylate AREB/ABF (ABA responsive element-binding protein/ABRE-binding factor) transcription factors (Liu et al. 2019) to participate in various biological processes, including seed germination and salt tolerance (Li et al. 2021b; Wang et al. 2020). Rice contains 10 SnRK2 members denoted as SAPK1-10 (stress-activated protein kinase) (Kobayashi et al. 2004; Liu et al. 2019), and *Ossapk1* mutants have been reported to exhibit reduced seed germination under salt stress (Lou et al. 2018). This result is consistent with the results that the seed germination and seedling growth in *Ossapk1* mutants were significantly reduced under salt stress compared to WT in our study.

In our study, *qNL3.1*, which was simultaneously identified by the four indices of seed germination under salt stress over the two years, is a key locus for seed germination under salt stress. Based on the classification of significant SNPs of *qNL3.1* according to Yano et al. (2016) and the expression levels of candidate genes, we hypothesized that *OsTTL* and *OsSAPK1* are both causal genes of *qNL3.1* that regulate rice seed germination under salt stress. However, since the genotype set for GWAS could not cover all the SNPs, it is possible that there are still other genes, whose SNPs are not included in the GWAS set, to regulate seed germination within the region of *qNL3.1*. To date, there are no reports that two or multiple genes have been identified to regulate seed germination at a locus, such as disease resistance-related genes (Deng et al. 2017; Chen et al. 2015). Previous reports showed that allantoin could activate the production of ABA, enhancing abiotic stress tolerance in plants (Watanabe et al. 2014; Takagi et al. 2016). *OsSAPK1*, an ABA-activated protein kinase (Lou et al. 2018), might be involved in ABA and its signaling pathways. It seems that there is crosstalk between *OsTTL* and *OsSAPK1* that regulates seed germination under salt stress. The regulatory mechanisms of *OsTTL* and *OsSAPK1* and their relationship or crosstalk in seed germination under salt stress should be studied in the future.

The genotypic selection strategy is superior to phenotypic selection in accelerating gene pyramiding by MAS (Xu et al. 2012). The optimal breeding scheme of gene pyramiding involves selecting a series of favorite target alleles after crossing base populations and pyramiding them into a single genotype (Servin et al. 2004; Xu et al. 2012). Among haplotype groups, the excellent Hap.1 of *OsTTL* and Hap.1 of *OsSAPK1* have higher contributions to seed germination under salt stress, which needs further confirmation via a transgenic approach. The combination of the excellent Hap.1 of *OsTTL* and Hap.1 of *OsSAPK1* could be used for gene pyramiding to develop salt-tolerant rice varieties with higher seed germination ability under salt stress. Moreover, six elite accessions, O-Luen-Cheung, Byakkoku Y 5006 Seln, Saturn, SLO 17, KPF-16 and Kiang-Chou-Chiu, carrying the combination of Hap.1 of *OsTTL* and Hap.1 of *OsSAPK1*, with high seed germination under salt stress in rice, would be useful in future rice breeding programs. Moreover, the physical positions between the *OsTTL* and *OsSAPK1* genes, both located within *qNL3.1,* were close, within approximately 19 kb on chromosome 3. Thereby, the excellent alleles of *OsTTL* and *OsSAPK1* genes, as a combination, could be quickly pyramided with other cloned excellent genes into one cultivar with reduced rounds of crossing in rice breeding.

## Conclusion

In this study, we identified a total of 60 loci significantly associated with seed germination under salt stress via a genome-wide association study, including ten loci identified in two years. Of these loci, the key locus *qNL3.1* was simultaneously identified with the four indices in the two years, and its two causal genes, *OsTTL* and *OsSAPK1*, positively regulated rice seed germination under stress. The excellent Hap.1 of *OsTTL* and Hap.1 of *OsSAPK1* were also found, and their combination could result in high seed germination under salt stress. Six accessions carrying the combination of Hap.1 of *OsTTL* and Hap.1 of *OsSAPK1* were identified with elite performance of seed germination under salt stress. Our identified loci and elite accessions might be applicable to improve rice seed germination under salt stress in the future.

## Supplementary Information

Below is the link to the electronic supplementary material.

Supplementary file1 (DOC 5878 KB)

## Data Availability

All data supporting the findings of this study are available within the paper and within its supplementary data published online.
