# Supplementary material for: Identification of a key locus, qNL3.1, associated with seed germination under salt stress via a genome-wide association study in rice
Source: Theor Appl Genet. 2023 Mar 13;136(3):58. doi: 10.1007/s00122-023-04252-x (PMC10011300; doi:10.1007/s00122-023-04252-x)
Supplement: Supplementary file 1 — Supplementary file1 (DOC 5878 KB) [file 122_2023_4252_MOESM1_ESM.doc]

# Supplementary Information


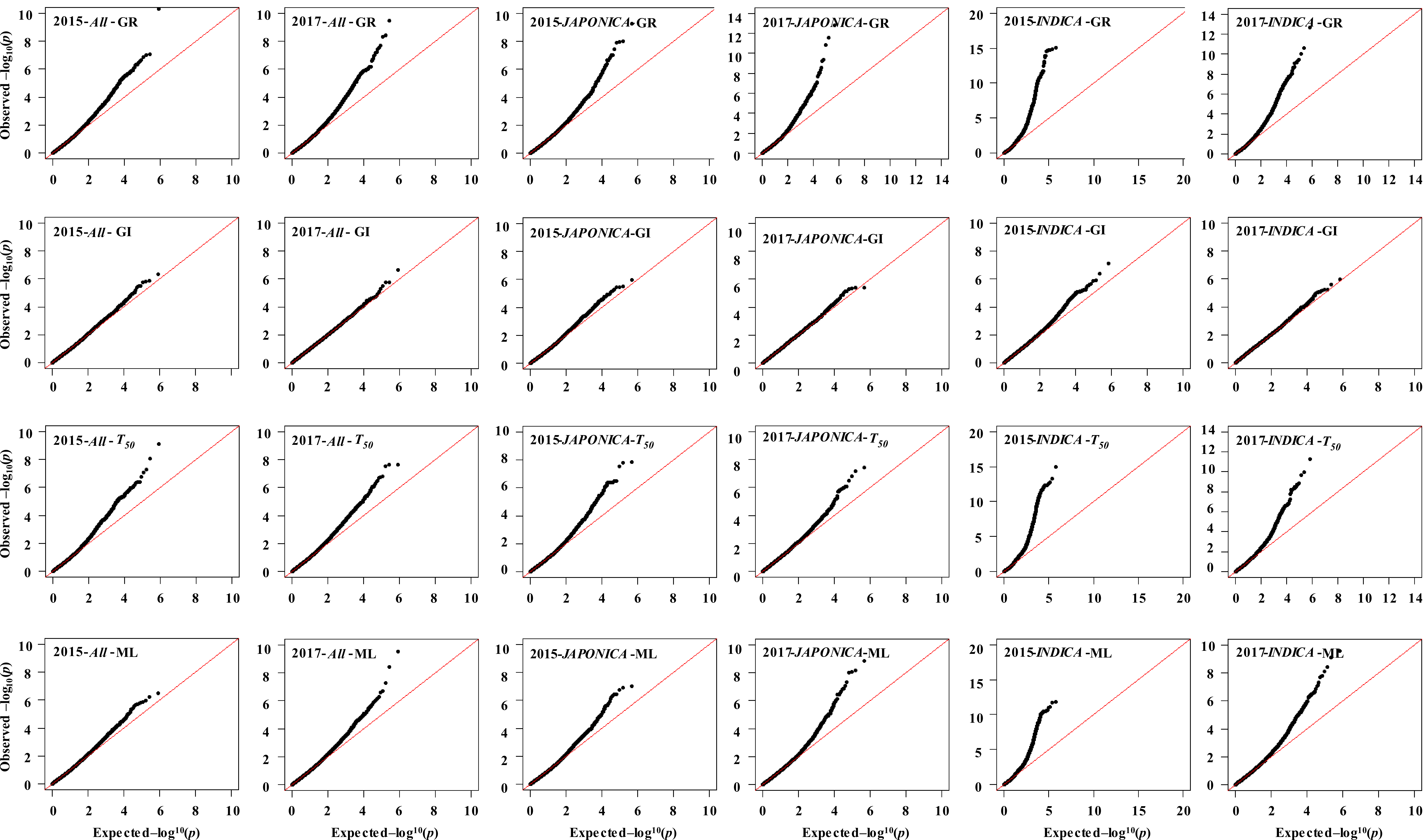


**Fig. S1** Quantile‒quantile plots for GR, GI, *T_50_* and ML under salt stress.


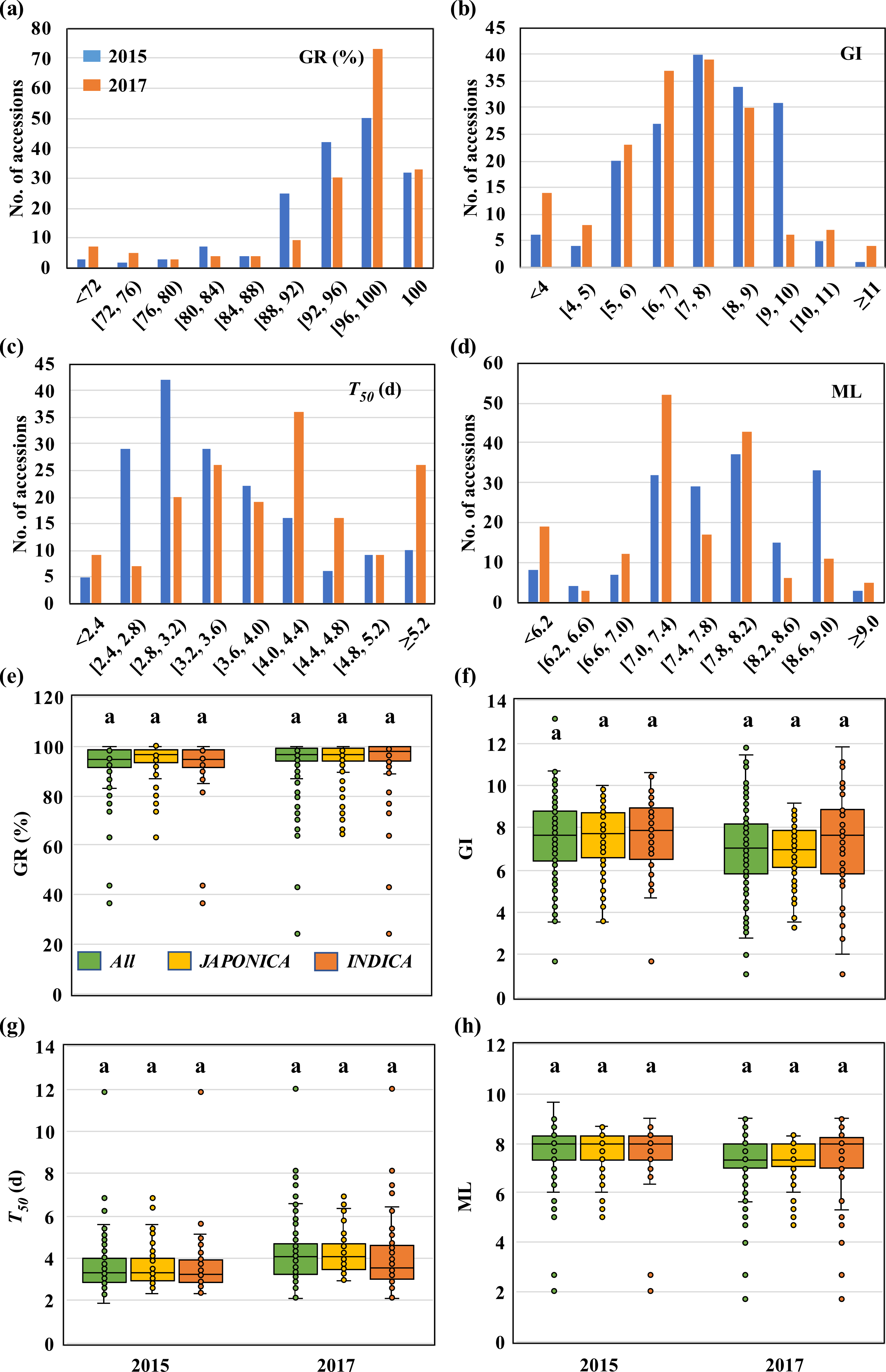


**Fig. S2** Phenotypic variation and distribution of GR, GI, *T_50_* and ML in RDP1. Histograms showing the distributions of GR (a), GI (b), *T_50_* (c) and ML (d) in 2015 and 2017. Boxplots of GR (e), GI (f), *T_50_* (g) and ML (h) in the *All*, *JAPONICA* and *INDICA* subgroups in 2015 and 2017. The *P* values were calculated using Student’s *t* test. Different lowercase letters indicate significant differences at the 0.05 level.


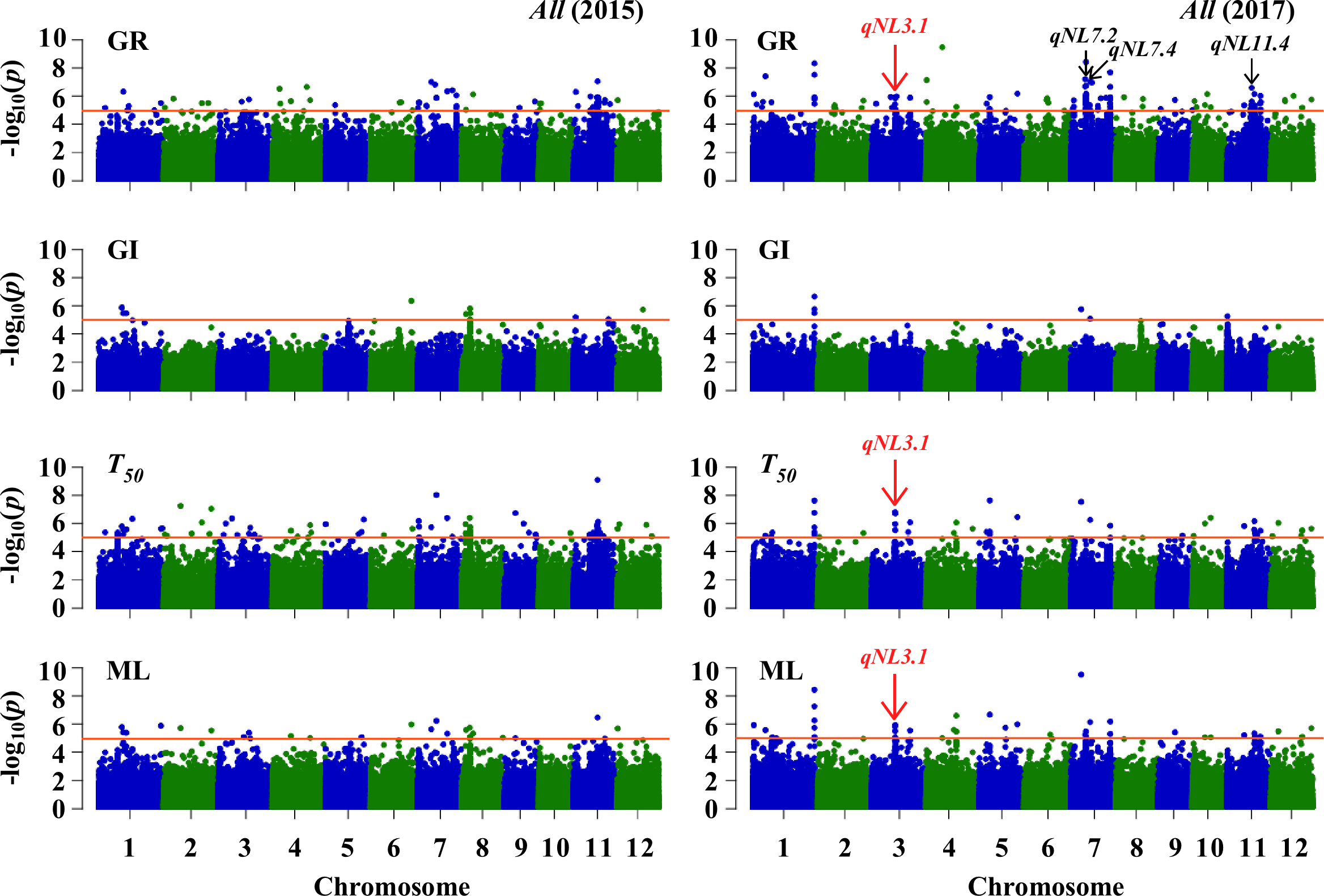


**Fig. S3** Genome-wide association analysis of GR, GI, *T_50_* and ML in *All*. Manhattan plots of GR, GI, *T_50_* and ML in 2015 (left) and 2017 (right). The horizontal red lines indicate the statistical significance threshold of *P*< 1×10^-5^. The arrows indicate the loci identified in both years.


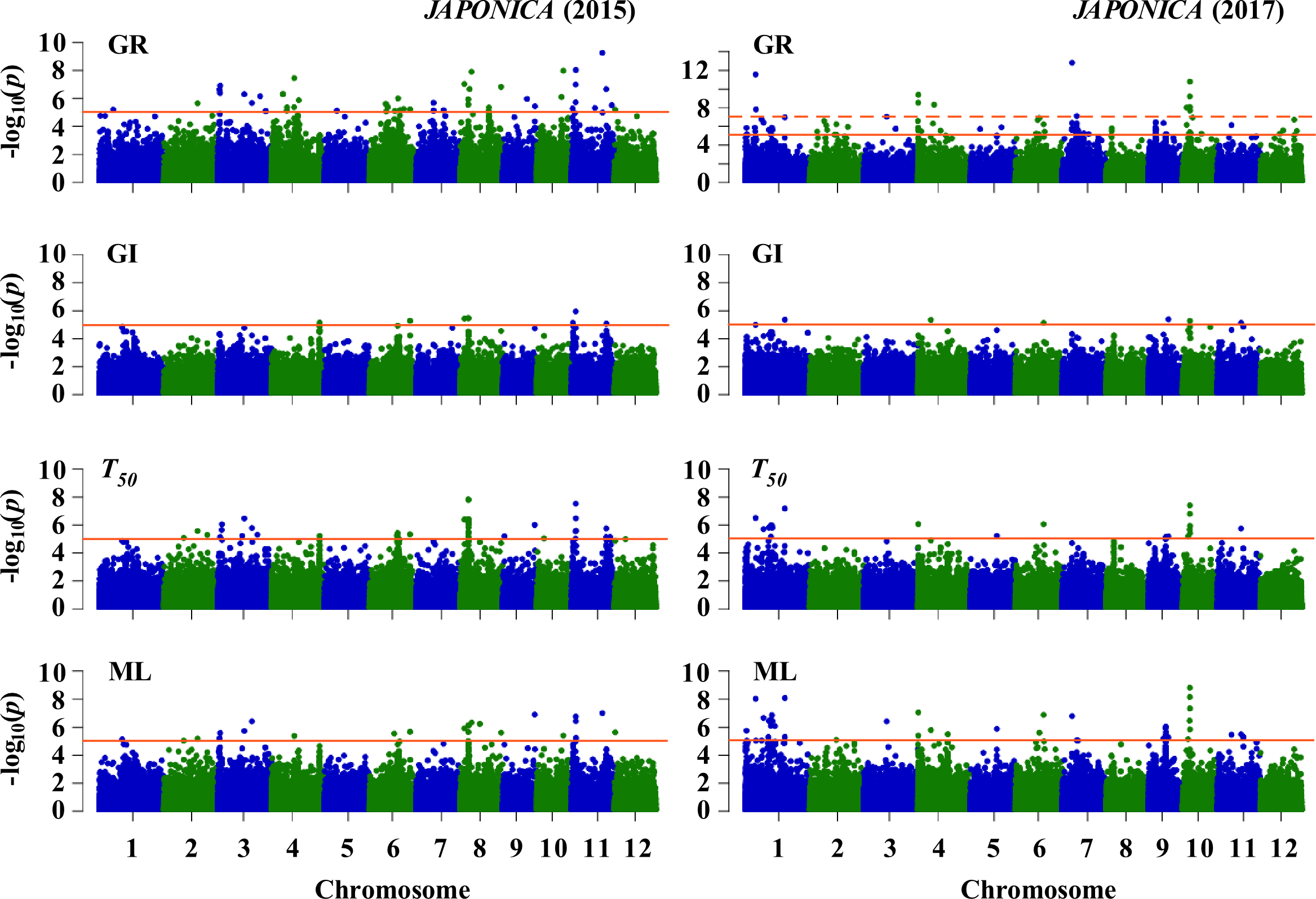


**Fig. S4** Genome-wide association analysis of GR, GI, *T_50_* and ML in *JAPONICA*. Manhattan plots of GR, GI, *T_50_* and ML in 2015 (left) and 2017 (right). The horizontal red solid lines indicate the statistical significance threshold of *P*< 1×10^-5^. The horizontal red dotted lines indicate the Bonferroni-corrected threshold of *P*< 1×10^-7^.


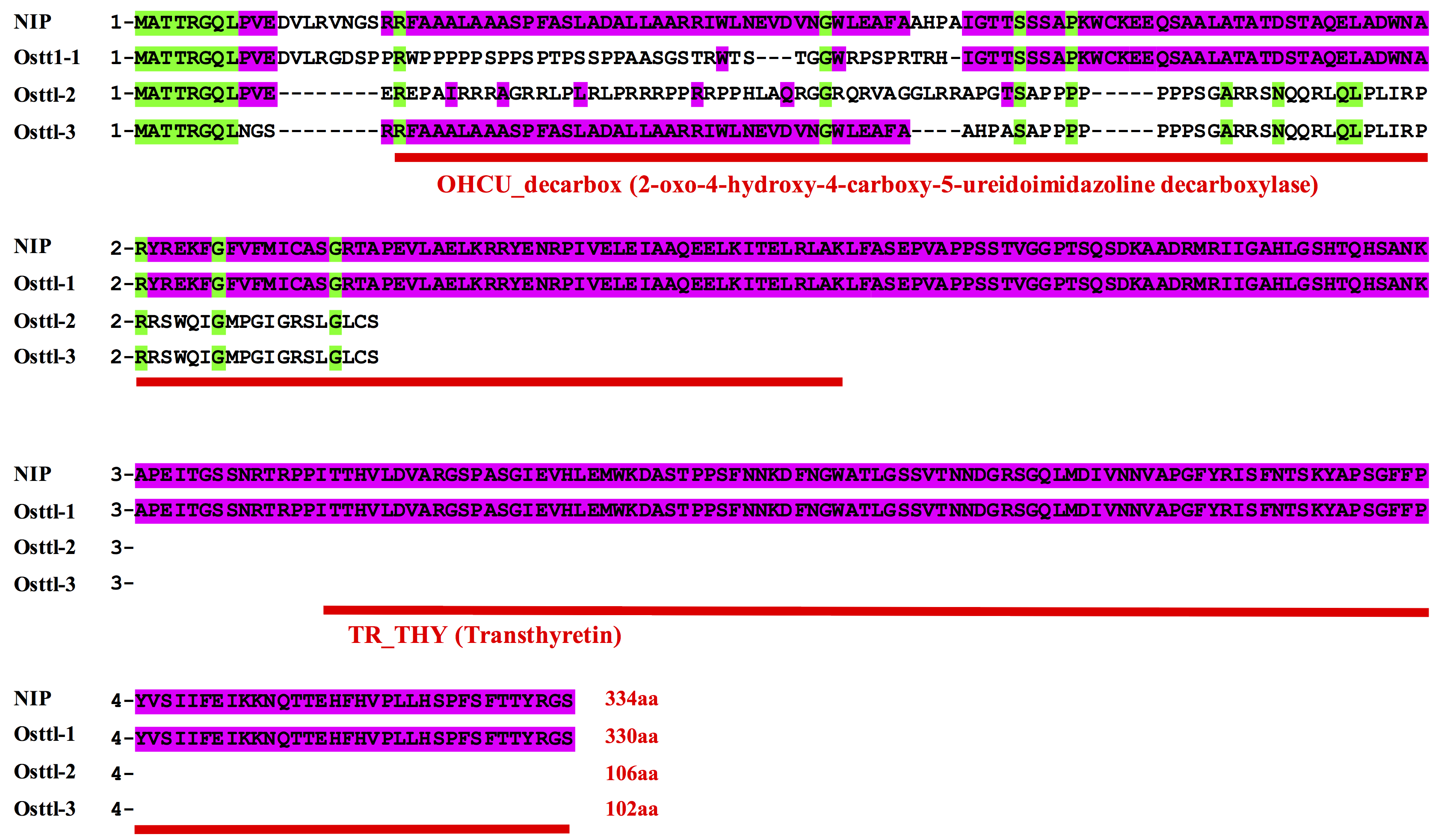


**Fig. S5** Comparison of amino acid sequences between WT and *Osttl* mutants. The red lines indicate OHCU_decarbox (2-oxo-4-hydroxy-4-carboxy-5-ureidoimidazoline decarboxylase) and TR_THY (transthyretin).


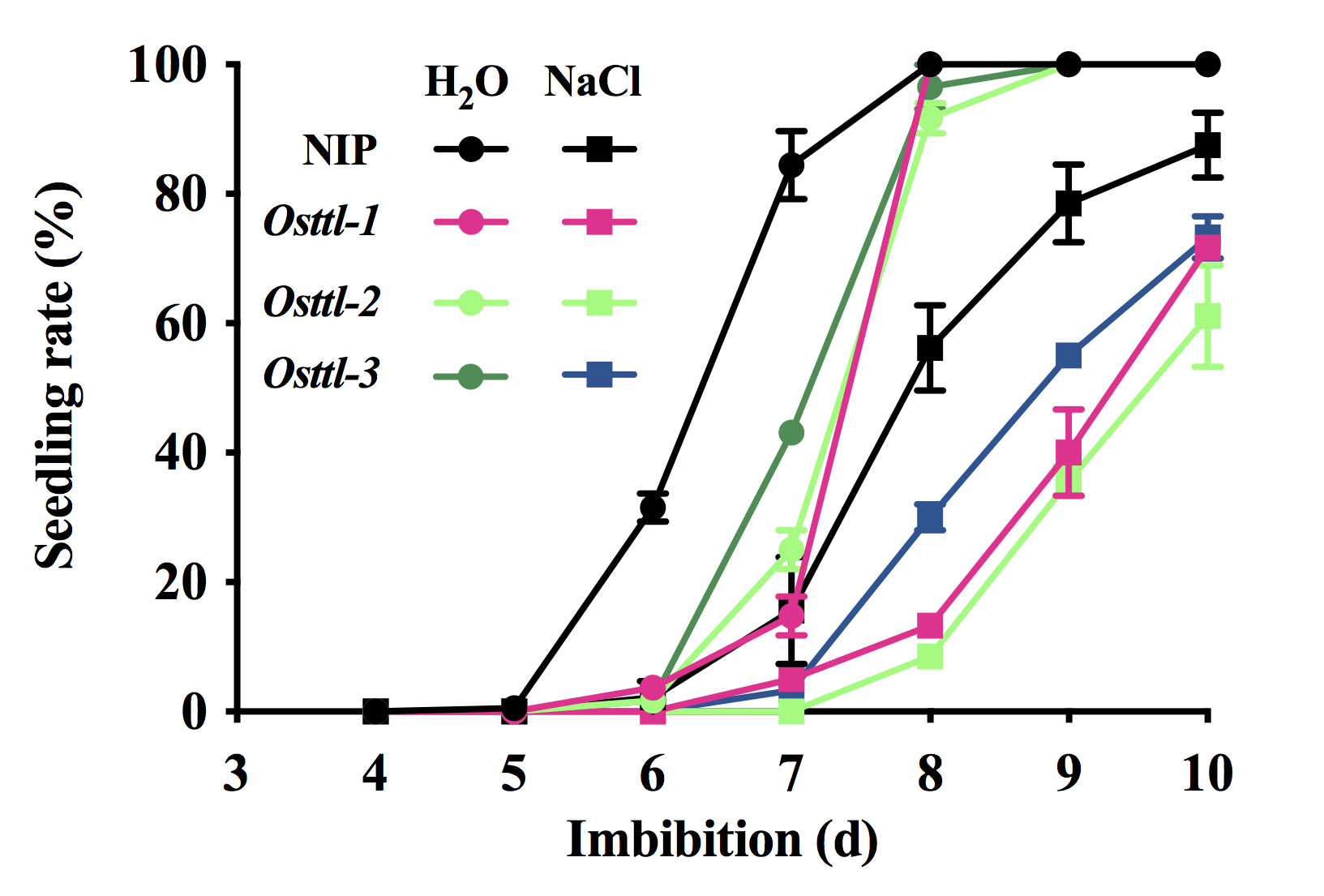


**Fig. S6** Dynamic changes in the seedling rates of WT and *Osttl* mutants 3-10 d after imbibition.


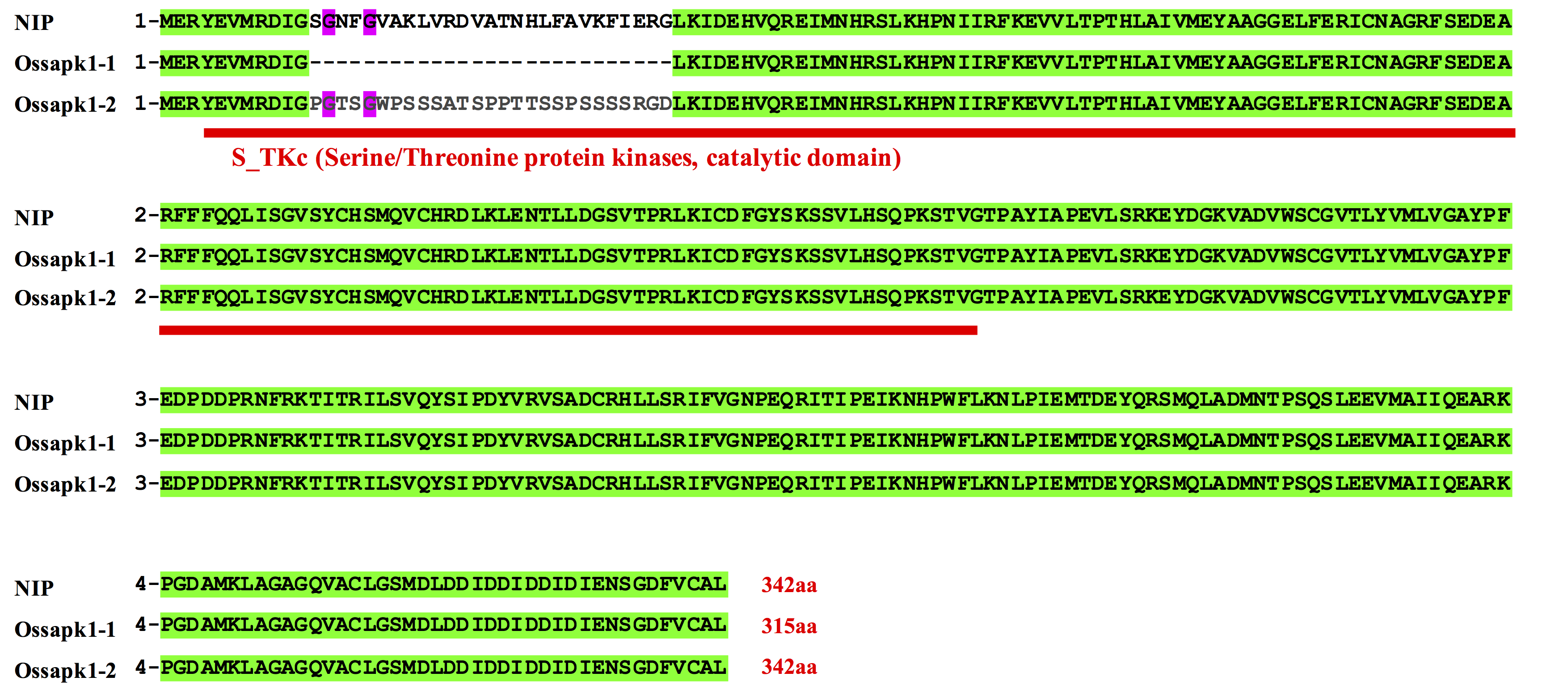


**Fig. S7** Comparison of amino acid sequences between WT and *Ossapk1* mutants. The red lines indicate serine/threonine protein kinases (S_Tke, catalytic domain).


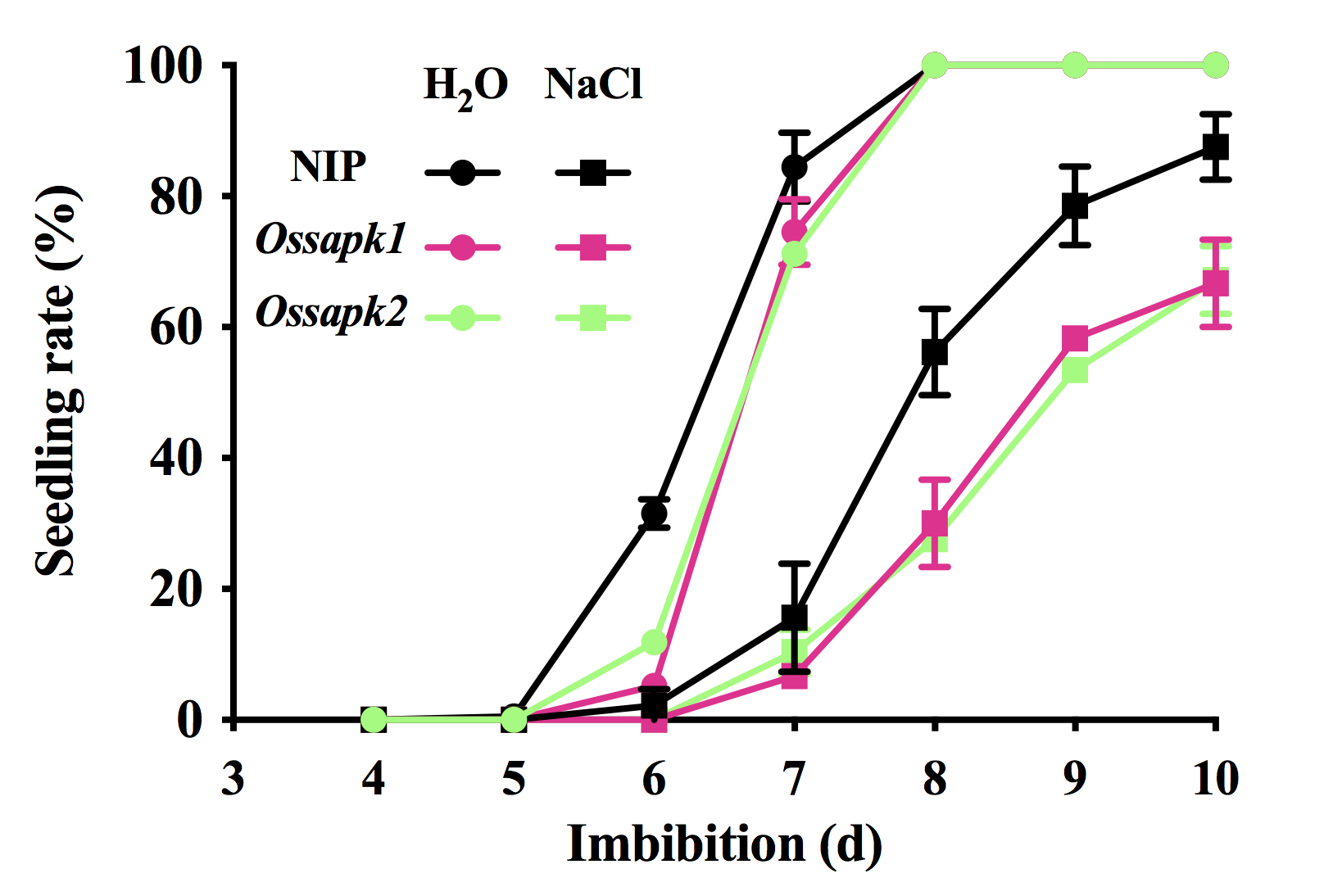


**Fig. S8** Dynamic changes in the seedling rates of WT and *Ossapk1* mutants at 3-10 d after imbibition.

**Table S1** Seed germination information of the 200 rice accessions for the association analysis.

| NSFTV ID. | Subgroups | Accession names | Country of origin | Mean GR _5d (%) of two years under H_2_O condition | 200 mM NaCl | | | | | | | | | Use |
| --- | --- | --- | --- | --- | --- | --- | --- | --- | --- | --- | --- | --- | --- | --- |
|  |  |  |  |  | 2015 | | | |  | 2017 | | | |  |
|  |  |  |  |  | GR (%) | T_50_ (d) | GI | ML |  | GR (%) | T_50_ (d) | GI | ML |  |
| NSFTV3 | IND | Ai-Chiao-Hong | China | 97.50 | 95.00 | 2.81 | 8.78 | 8.33 |  | 95.92 | 3.26 | 7.86 | 8.00 | GWAS |
| NSFTV4 | AUS | NSF-TV 4 | India | 98.17 | 90.83 | 4.46 | 5.81 | 7.00 |  | 89.00 | 4.76 | 5.22 | 6.67 | GWAS |
| NSFTV5 | AROMATIC | NSF-TV 5 | India | 99.17 | 80.00 | 6.21 | 3.87 | 5.33 |  | 91.48 | 4.77 | 5.65 | 7.00 | GWAS |
| NSFTV6 | AUS | ARC 7229 | India | 97.00 | 81.67 | 4.92 | 5.43 | 6.67 |  | 77.00 | 6.23 | 4.21 | 5.33 | GWAS |
| NSFTV7 | TRJ | Arias | Indonesia | 97.50 | 78.33 | 4.30 | 5.47 | 6.33 |  | 99.00 | 4.73 | 5.73 | 7.00 | GWAS |
| NSFTV15 | TEJ | Beonjo | South Korea | 100.00 | 95.00 | 3.07 | 8.18 | 8.00 |  | 100.00 | 3.89 | 7.61 | 8.00 | GWAS |
| NSFTV16 | AROMATIC | Bico Branco | Brazil | 96.67 | 95.00 | 4.81 | 5.86 | 7.00 |  | 77.28 | 6.95 | 3.49 | 5.33 | GWAS |
| NSFTV17 | IND | Binulawan | Philippines | 96.50 | 98.33 | 2.98 | 8.51 | 8.33 |  | 97.00 | 3.63 | 7.17 | 7.67 | GWAS |
| NSFTV21 | IND | Byakkoku Y 5006 Seln | Australia | 98.33 | 100.00 | 2.69 | 9.43 | 8.67 |  | 100.00 | 2.17 | 11.26 | 9.00 | GWAS |
| NSFTV25 | TRJ | Carolina Gold | United States | 99.14 | 100.00 | 2.69 | 9.83 | 8.67 |  | 100.00 | 3.01 | 9.13 | 8.33 | GWAS |
| NSFTV26 | TRJ | Carolina Gold Sel | United States | 95.99 | 96.67 | 4.73 | 5.46 | 7.00 |  | 98.98 | 2.90 | 8.84 | 8.33 | GWAS |
| NSFTV29 | IND | Chau | Vietnam | 100.00 | 95.00 | 3.61 | 7.09 | 7.67 |  | 98.98 | 3.24 | 8.11 | 8.00 | GWAS |
| NSFTV30 | IND | Chiem Chanh | Vietnam | 99.17 | 100.00 | 2.71 | 9.53 | 8.67 |  | 100.00 | 3.25 | 8.95 | 8.00 | GWAS |
| NSFTV31 | TEJ | Chinese | China | 98.33 | 98.33 | 2.58 | 9.80 | 8.67 |  | 100.00 | 3.34 | 7.74 | 8.00 | GWAS |
| NSFTV32 | TEJ | Chodongji | South Korea | 97.44 | 98.33 | 2.81 | 9.20 | 8.67 |  | 93.10 | 3.53 | 7.94 | 8.00 | GWAS |
| NSFTV33 | AUS | Chuan 4 | Taiwan | 95.82 | 43.33 | 11.81 | 1.87 | 2.67 |  | 43.00 | 12.00 | 1.99 | 2.67 | GWAS |
| NSFTV36 | TEJ | CS-M3 | United States-CA | 95.67 | 63.33 | 6.95 | 3.59 | 5.00 |  | 90.10 | 5.77 | 4.47 | 6.33 | GWAS |
| NSFTV41 | ADMIX | Darmali | Nepal | 98.33 | 91.67 | 4.24 | 6.01 | 7.33 |  | 88.10 | 4.31 | 5.67 | 6.67 | GWAS |
| NSFTV43 | IND | Dee Geo Woo Gen | Taiwan | 97.21 | 100.00 | 2.54 | 9.96 | 8.67 |  | 96.59 | 3.56 | 7.60 | 8.00 | GWAS |
| NSFTV44 | AUS | Dhala Shaitta | Bangladesh | 100.00 | 96.67 | 2.93 | 8.39 | 8.33 |  | 100.00 | 2.86 | 8.78 | 8.33 | GWAS |
| NSFTV45 | AROMATIC | Dom-sufid | Iran | 99.17 | 100.00 | 4.51 | 5.94 | 7.00 |  | 81.76 | 5.61 | 4.33 | 6.00 | GWAS |
| NSFTV46 | TRJ | Dourado Agulha | Brazil | 99.17 | 93.33 | 2.79 | 9.00 | 8.33 |  | 98.98 | 3.16 | 8.28 | 8.00 | GWAS |
| NSFTV51 | TEJ | Early Wataribune | Japan | 99.17 | 100.00 | 2.90 | 9.06 | 8.67 |  | 100.00 | 4.24 | 7.12 | 7.33 | GWAS |
| NSFTV53 | AROMATIC | Firooz | Iran | 98.25 | 98.33 | 3.53 | 7.33 | 7.67 |  | 96.67 | 4.13 | 7.08 | 7.33 | GWAS |
| NSFTV55 | ADMIX | Gerdeh | Iran | 96.67 | 95.00 | 5.03 | 5.25 | 6.67 |  | 87.09 | 5.24 | 4.88 | 6.33 | GWAS |
| NSFTV65 | TRJ | Honduras | Honduras | 99.49 | 98.33 | 2.80 | 9.20 | 8.67 |  | 96.96 | 3.02 | 8.40 | 8.00 | GWAS |
| NSFTV69 | TRJ | IAC 25 | Brazil | 99.50 | 98.33 | 2.72 | 9.25 | 8.67 |  | 97.98 | 3.26 | 8.01 | 8.00 | GWAS |
| NSFTV70 | TRJ | Iguape Cateto | Haiti | 100.00 | 95.00 | 3.23 | 7.78 | 8.00 |  | 100.00 | 3.72 | 8.09 | 8.00 | GWAS |
| NSFTV72 | IND | IR 8 | Philippines | 100.00 | 100.00 | 3.19 | 8.23 | 8.00 |  | 100.00 | 3.47 | 7.66 | 8.00 | GWAS |
| NSFTV73 | TRJ | IRAT 177 | French Guiana | 99.50 | 93.33 | 3.13 | 7.87 | 8.00 |  | 98.00 | 3.89 | 6.95 | 7.67 | GWAS |
| NSFTV74 | IND | IRGA 409 | Brazil | 96.15 | 95.00 | 2.75 | 9.09 | 8.67 |  | 98.96 | 3.69 | 7.28 | 7.67 | GWAS |
| NSFTV75 | TRJ | Jambu | Indonesia | 95.80 | 95.83 | 3.18 | 8.00 | 8.00 |  | 96.36 | 4.40 | 6.36 | 7.33 | GWAS |
| NSFTV76 | IND | Jaya | India | 99.49 | 98.33 | 3.32 | 7.83 | 8.00 |  | 98.98 | 4.01 | 6.86 | 7.33 | GWAS |
| NSFTV78 | AUS | Jhona 349 | India | 99.17 | 90.00 | 3.11 | 7.61 | 7.67 |  | 96.55 | 3.47 | 8.10 | 8.00 | GWAS |
| NSFTV83 | TEJ | Kamenoo | Japan | 97.29 | 91.67 | 4.12 | 6.25 | 7.33 |  | 75.74 | 6.45 | 3.84 | 5.33 | GWAS |
| NSFTV90 | IND | Kiang-Chou-Chiu | Taiwan | 97.70 | 100.00 | 2.39 | 10.43 | 8.67 |  | 97.98 | 2.56 | 9.87 | 8.67 | GWAS |
| NSFTV94 | TEJ | Koshihikari | Japan | 100.00 | 83.33 | 6.35 | 3.79 | 5.67 |  | 96.55 | 5.16 | 5.43 | 6.67 | GWAS |
| NSFTV98 | TRJ | L-202 | United States_CA | 99.44 | 100.00 | 2.91 | 8.75 | 8.33 |  | 93.79 | 3.98 | 6.45 | 7.67 | GWAS |
| NSFTV99 | TRJ | LAC 23 | Liberia | 97.50 | 100.00 | 3.44 | 7.69 | 8.00 |  | 94.94 | 3.69 | 7.07 | 7.67 | GWAS |
| NSFTV100 | ADMIX | Lacrosse | United States | 98.33 | 100.00 | 2.39 | 10.64 | 9.00 |  | 100.00 | 3.39 | 8.39 | 8.00 | GWAS |
| NSFTV102 | IND | Leung Pratew | Thailand | 96.50 | 100.00 | 3.12 | 8.29 | 8.00 |  | 94.00 | 3.18 | 7.76 | 8.00 | GWAS |
| NSFTV103 | TEJ | Luk Takhar | Afghanistan | 99.17 | 95.00 | 3.01 | 8.28 | 8.00 |  | 89.83 | 4.76 | 5.83 | 6.67 | GWAS |
| NSFTV105 | AUS | Mehr | Iran | 99.17 | 96.67 | 3.73 | 7.01 | 7.67 |  | 94.00 | 5.10 | 5.23 | 6.67 | GWAS |
| NSFTV108 | TRJ | Moroberekan | Guinea | 97.83 | 98.33 | 3.73 | 7.08 | 7.67 |  | 100.00 | 4.05 | 6.65 | 7.33 | GWAS |
| NSFTV116 | TRJ | NSF-TV 116 | Pakistan | 98.33 | 91.67 | 3.07 | 8.18 | 8.00 |  | 98.33 | 3.75 | 7.83 | 8.00 | GWAS |
| NSFTV117 | IND | O-Luen-Cheung | Taiwan | 97.33 | 100.00 | 2.32 | 10.63 | 9.00 |  | 98.98 | 2.26 | 11.10 | 9.00 | GWAS |
| NSFTV120 | TRJ | OS6 | Nigeria | 100.00 | 100.00 | 3.29 | 7.93 | 8.00 |  | 100.00 | 4.31 | 6.28 | 7.33 | GWAS |
| NSFTV121 | TEJ | Ostiglia | Argentina | 99.17 | 78.21 | 4.47 | 5.48 | 6.33 |  | 96.96 | 4.89 | 5.51 | 7.00 | GWAS |
| NSFTV122 | TRJ | Padi Kasalle | Indonesia | 98.33 | 90.00 | 3.42 | 7.09 | 7.33 |  | 96.30 | 4.10 | 6.95 | 7.33 | GWAS |
| NSFTV123 | IND | Pagaiyahan | Taiwan | 99.17 | 100.00 | 2.86 | 9.43 | 8.67 |  | 100.00 | 2.35 | 10.25 | 8.67 | GWAS |
| NSFTV124 | AROMATIC | Pankhari 203 | India | 99.17 | 100.00 | 2.70 | 9.48 | 8.67 |  | 100.00 | 2.69 | 9.64 | 8.67 | GWAS |
| NSFTV125 | IND | Pao-Tou-Hung | China | 100.00 | 96.67 | 3.17 | 8.00 | 8.00 |  | 100.00 | 3.48 | 8.31 | 8.00 | GWAS |
| NSFTV126 | IND | Pappaku | Taiwan | 95.83 | 95.00 | 4.30 | 5.87 | 7.00 |  | 99.00 | 4.30 | 6.30 | 7.33 | GWAS |
| NSFTV129 | IND | Peh-Kuh | Taiwan | 100.00 | 96.67 | 2.92 | 8.44 | 8.33 |  | 100.00 | 2.26 | 10.15 | 8.67 | GWAS |
| NSFTV130 | IND | Peh-Kuh-Tsao-Tu | Taiwan | 100.00 | 96.67 | 2.99 | 8.54 | 8.33 |  | 100.00 | 2.69 | 10.06 | 8.67 | GWAS |
| NSFTV134 | TEJ | Romeo | Italy | 95.00 | 83.33 | 3.40 | 6.87 | 7.33 |  | 82.56 | 4.23 | 6.41 | 7.00 | GWAS |
| NSFTV137 | IND | RTS14 | Vietnam | 95.65 | 88.33 | 2.99 | 8.72 | 8.00 |  | 100.00 | 2.94 | 8.56 | 8.33 | GWAS |
| NSFTV140 | ADMIX | Saturn | United States | 100.00 | 95.00 | 2.51 | 9.46 | 8.67 |  | 100.00 | 2.17 | 11.43 | 9.00 | GWAS |
| NSFTV143 | TEJ | Shinriki | Japan | 96.67 | 98.33 | 2.90 | 8.63 | 8.33 |  | 93.22 | 5.07 | 5.17 | 6.67 | GWAS |
| NSFTV150 | TRJ | Sultani | Egypt | 98.33 | 91.67 | 2.64 | 9.01 | 8.67 |  | 97.92 | 3.26 | 7.96 | 8.00 | GWAS |
| NSFTV151 | TEJ | Suweon | Korea | 99.11 | 100.00 | 2.78 | 8.96 | 8.33 |  | 93.10 | 4.29 | 6.29 | 7.33 | GWAS |
| NSFTV152 | AUS | T 1 | India | 100.00 | 85.00 | 3.92 | 6.27 | 7.33 |  | 95.00 | 4.07 | 7.02 | 7.33 | GWAS |
| NSFTV155 | TEJ | Ta Mao Tsao | China | 97.44 | 98.33 | 5.43 | 5.14 | 6.67 |  | 73.05 | 6.89 | 3.27 | 5.33 | GWAS |
| NSFTV157 | TEJ | Tainan Iku 487 | Taiwan | 99.17 | 98.33 | 2.65 | 9.52 | 8.67 |  | 95.00 | 3.94 | 7.36 | 7.67 | GWAS |
| NSFTV171 | IND | ZHE 733 | China | 99.48 | 95.00 | 3.17 | 7.91 | 8.00 |  | 98.96 | 3.92 | 7.45 | 7.67 | GWAS |
| NSFTV177 | TEJ | 68-2 | France | 100.00 | 98.33 | 3.65 | 7.23 | 7.67 |  | 100.00 | 4.75 | 6.28 | 7.33 | GWAS |
| NSFTV178 | AUS | ARC 6578 | India | 95.17 | 86.67 | 3.98 | 6.28 | 7.33 |  | 81.67 | 4.09 | 6.37 | 7.00 | GWAS |
| NSFTV179 | TEJ | Bellardone | France | 96.14 | 94.33 | 3.48 | 7.33 | 7.67 |  | 94.37 | 4.88 | 5.68 | 7.00 | GWAS |
| NSFTV180 | TEJ | Benllok | Peru | 99.17 | 96.67 | 3.66 | 7.07 | 7.67 |  | 98.00 | 4.37 | 6.53 | 7.33 | GWAS |
| NSFTV182 | ADMIX | Blue Rose Supreme | United States | 99.17 | 88.33 | 3.11 | 8.32 | 7.67 |  | 94.37 | 4.32 | 6.55 | 7.33 | GWAS |
| NSFTV183 | TRJ | Boa Vista | El Salvador | 94.94 | 90.00 | 3.31 | 7.36 | 7.33 |  | 96.67 | 3.49 | 8.08 | 8.00 | GWAS |
| NSFTV185 | TRJ | British Honduras Creole | Belize | 99.44 | 100.00 | 3.04 | 8.50 | 8.00 |  | 98.84 | 3.78 | 7.19 | 7.67 | GWAS |
| NSFTV186 | TEJ | Bul Zo | South Korea | 99.17 | 88.33 | 3.94 | 6.43 | 7.33 |  | 94.77 | 3.63 | 7.79 | 8.00 | GWAS |
| NSFTV189 | IND | Criollo La Fria | Venezuela | 97.50 | 93.33 | 2.56 | 9.75 | 8.67 |  | 97.98 | 2.06 | 11.80 | 9.00 | GWAS |
| NSFTV191 | AROMATIC | Dom Zard | Iran | 98.33 | 98.33 | 4.85 | 5.59 | 7.00 |  | 75.86 | 7.79 | 3.02 | 5.00 | GWAS |
| NSFTV192 | TEJ | Erythroceros Hokkaido | Poland | 98.33 | 93.33 | 3.52 | 7.24 | 7.67 |  | 95.90 | 3.47 | 7.44 | 7.67 | GWAS |
| NSFTV195 | TRJ | IRAT 13 | Cote D'Ivoire | 95.71 | 96.67 | 3.07 | 8.18 | 8.00 |  | 100.00 | 4.59 | 6.24 | 7.33 | GWAS |
| NSFTV198 | TRJ | Leah | Bulgaria | 96.67 | 100.00 | 3.13 | 8.35 | 8.00 |  | 94.94 | 5.09 | 5.26 | 6.67 | GWAS |
| NSFTV201 | TRJ | Pate Blanc Mn 1 | Cote D'Ivoire | 99.17 | 90.00 | 3.24 | 7.66 | 7.67 |  | 95.00 | 4.23 | 6.71 | 7.33 | GWAS |
| NSFTV205 | ADMIX | Rinaldo Bersani | Italy | 100.00 | 93.33 | 2.87 | 8.64 | 8.33 |  | 96.15 | 3.76 | 7.01 | 7.67 | GWAS |
| NSFTV208 | IND | SLO 17 | India | 99.17 | 98.33 | 2.59 | 9.74 | 8.67 |  | 98.28 | 2.32 | 10.86 | 9.00 | GWAS |
| NSFTV209 | IND | Tchibanga | Gabon | 95.00 | 96.67 | 3.26 | 7.83 | 8.00 |  | 64.71 | 7.07 | 3.38 | 4.67 | GWAS |
| NSFTV213 | TRJ | WC 3397 | Jamaica | 100.00 | 98.33 | 3.97 | 6.61 | 7.67 |  | 93.33 | 3.60 | 6.83 | 7.67 | GWAS |
| NSFTV214 | TRJ | WC 4419 | Honduras | 100.00 | 93.33 | 3.34 | 7.68 | 8.00 |  | 100.00 | 4.05 | 7.17 | 7.33 | GWAS |
| NSFTV215 | TRJ | WC 4443 | Bolivia | 95.41 | 93.33 | 3.97 | 6.59 | 7.67 |  | 92.28 | 4.19 | 6.49 | 7.33 | GWAS |
| NSFTV218 | ADMIX | PI 298967-1 | Australia | 98.30 | 90.00 | 3.84 | 6.43 | 7.33 |  | 98.33 | 4.19 | 7.03 | 7.33 | GWAS |
| NSFTV221 | AROMATIC | Sadri Belyi | Azerbaijan | 98.33 | 95.00 | 4.08 | 6.34 | 7.33 |  | 100.00 | 4.14 | 6.43 | 7.33 | GWAS |
| NSFTV222 | IND | Paraiba Chines Nova | Brazil | 99.17 | 93.33 | 3.76 | 6.90 | 7.67 |  | 100.00 | 3.01 | 8.80 | 8.00 | GWAS |
| NSFTV226 | TRJ | IRAT 44 | Burkina Faso | 98.67 | 96.67 | 3.97 | 6.70 | 7.67 |  | 100.00 | 3.52 | 7.64 | 8.00 | GWAS |
| NSFTV227 | ADMIX | Riz Local | Burkina Faso | 99.17 | 95.00 | 3.64 | 7.06 | 7.67 |  | 85.00 | 4.10 | 6.56 | 7.00 | GWAS |
| NSFTV228 | AUS | CA 902/B/2/1 | Chad | 98.98 | 91.67 | 3.61 | 6.96 | 7.67 |  | 98.98 | 3.63 | 7.24 | 7.67 | GWAS |
| NSFTV232 | TEJ | Shangyu 394 | China | 95.04 | 93.33 | 5.18 | 5.04 | 6.67 |  | 85.93 | 6.51 | 4.39 | 5.67 | GWAS |
| NSFTV233 | TEJ | Sung Liao 2 | China | 95.33 | 100.00 | 4.19 | 6.49 | 7.33 |  | 70.56 | 6.58 | 3.51 | 5.33 | GWAS |
| NSFTV236 | ADMIX | WC 521 | China | 100.00 | 98.33 | 4.83 | 5.73 | 7.00 |  | 96.67 | 6.02 | 4.55 | 6.33 | GWAS |
| NSFTV239 | TRJ | WAB 502-13-4-1 | Cote D'Ivoire | 98.28 | 96.67 | 3.32 | 7.74 | 8.00 |  | 98.15 | 4.27 | 6.99 | 7.33 | GWAS |
| NSFTV242 | TRJ | 27.00 | Dominican Republic | 96.67 | 96.67 | 2.96 | 8.51 | 8.33 |  | 98.33 | 4.20 | 6.89 | 7.33 | GWAS |
| NSFTV245 | TEJ | Sab Ini | Egypt | 97.50 | 93.33 | 4.03 | 6.33 | 7.33 |  | 96.30 | 5.00 | 5.67 | 7.00 | GWAS |
| NSFTV247 | TEJ | Desvauxii | Former Soviet Union | 96.44 | 98.33 | 3.07 | 8.58 | 8.00 |  | 100.00 | 4.36 | 7.00 | 7.33 | GWAS |
| NSFTV252 | IND | Djimoron | Guinea | 100.00 | 98.33 | 2.77 | 9.77 | 8.67 |  | 98.96 | 2.98 | 8.55 | 8.33 | GWAS |
| NSFTV255 | IND | Pai Hok Glutinous | Hong Kong | 98.33 | 95.00 | 2.81 | 9.14 | 8.67 |  | 100.00 | 2.66 | 10.11 | 8.67 | GWAS |
| NSFTV257 | TEJ | Agusita | Hungary | 98.33 | 100.00 | 2.70 | 9.62 | 8.67 |  | 86.38 | 3.40 | 7.87 | 7.67 | GWAS |
| NSFTV259 | ADMIX | Sadri Tor Misri | Iran | 95.00 | 83.33 | 3.84 | 6.54 | 7.33 |  | 98.33 | 3.42 | 8.30 | 8.00 | GWAS |
| NSFTV262 | AUS | Halwa Gose Red | Iraq | 95.17 | 36.67 | 12.00 | 1.67 | 2.00 |  | 24.00 | 12.00 | 1.04 | 1.67 | GWAS |
| NSFTV263 | TEJ | Maratelli | Italy | 98.68 | 76.67 | 5.56 | 4.65 | 6.00 |  | 94.44 | 4.20 | 6.66 | 7.33 | GWAS |
| NSFTV266 | ADMIX | Hiderisirazu | Japan | 95.00 | 90.00 | 2.84 | 8.74 | 8.00 |  | 96.55 | 3.04 | 8.27 | 8.00 | GWAS |
| NSFTV271 | ADMIX | M. Blatec | Macedonia | 97.50 | 90.00 | 4.05 | 6.03 | 7.00 |  | 93.28 | 5.22 | 5.03 | 6.67 | GWAS |
| NSFTV273 | ADMIX | Varyla | Madagascar | 98.33 | 98.33 | 3.40 | 7.70 | 8.00 |  | 98.33 | 3.94 | 6.81 | 7.67 | GWAS |
| NSFTV276 | AUS | Kaukau | Mali | 99.17 | 92.50 | 5.11 | 5.03 | 6.67 |  | 82.00 | 6.44 | 3.91 | 5.67 | GWAS |
| NSFTV277 | TEJ | Gambiaka Sebela | Mali | 98.30 | 95.00 | 4.08 | 6.70 | 7.33 |  | 100.00 | 4.13 | 7.18 | 7.33 | GWAS |
| NSFTV278 | ADMIX | C1-6-5-3 | Mexico | 100.00 | 85.00 | 5.11 | 4.84 | 6.33 |  | 96.67 | 4.58 | 6.03 | 7.33 | GWAS |
| NSFTV281 | TEJ | Patna | Morocco | 96.61 | 93.33 | 4.23 | 6.26 | 7.33 |  | 79.53 | 6.25 | 3.71 | 5.33 | GWAS |
| NSFTV283 | TEJ | Chibica | Mozambique | 97.38 | 100.00 | 4.16 | 6.41 | 7.33 |  | 96.43 | 4.56 | 6.07 | 7.33 | GWAS |
| NSFTV285 | TRJ | Tox 782-20-1 | Nigeria | 95.83 | 91.67 | 3.09 | 8.13 | 8.00 |  | 97.78 | 3.11 | 8.46 | 8.00 | GWAS |
| NSFTV288 | TEJ | Italica Carolina | Poland | 95.71 | 91.67 | 4.32 | 5.85 | 7.00 |  | 66.67 | 6.32 | 3.26 | 5.00 | GWAS |
| NSFTV290 | TEJ | Amposta | Puerto Rico | 98.67 | 98.33 | 3.63 | 7.21 | 7.67 |  | 64.61 | 7.01 | 3.74 | 4.67 | GWAS |
| NSFTV295 | TEJ | Bombilla | Spain | 98.33 | 75.00 | 3.84 | 6.38 | 7.00 |  | 91.33 | 4.26 | 6.77 | 7.33 | GWAS |
| NSFTV297 | TEJ | Bahia | Spain | 96.41 | 86.67 | 4.25 | 6.04 | 7.00 |  | 95.65 | 4.02 | 7.52 | 7.67 | GWAS |
| NSFTV300 | TEJ | Sml Kapuri | Suriname | 96.48 | 83.33 | 4.20 | 5.47 | 6.67 |  | 75.59 | 5.86 | 4.65 | 6.00 | GWAS |
| NSFTV304 | IND | 519.00 | Uruguay | 97.32 | 100.00 | 2.85 | 8.96 | 8.33 |  | 98.86 | 3.42 | 8.56 | 8.00 | GWAS |
| NSFTV308 | TRJ | Llanero 501 | Venezuela | 98.99 | 95.00 | 2.76 | 9.05 | 8.67 |  | 97.96 | 3.07 | 8.49 | 8.00 | GWAS |
| NSFTV309 | TRJ | Manzano | Zaire | 100.00 | 96.67 | 2.89 | 8.92 | 8.33 |  | 99.00 | 3.28 | 7.99 | 8.00 | GWAS |
| NSFTV311 | TEJ | 56-122-23 | Thailand | 100.00 | 93.33 | 3.49 | 7.04 | 7.67 |  | 98.33 | 4.26 | 6.91 | 7.33 | GWAS |
| NSFTV313 | IND | BR24 | Bangladesh | 97.63 | 96.67 | 3.21 | 7.97 | 8.00 |  | 98.00 | 3.08 | 8.32 | 8.00 | GWAS |
| NSFTV316 | AUS | DD 62 | Bangladesh | 100.00 | 100.00 | 3.08 | 8.49 | 8.00 |  | 99.02 | 3.43 | 7.71 | 8.00 | GWAS |
| NSFTV318 | AUS | DJ 24 | Bangladesh | 97.00 | 88.33 | 3.72 | 6.73 | 7.33 |  | 94.00 | 4.25 | 6.04 | 7.33 | GWAS |
| NSFTV319 | AUS | DK 12 | Bangladesh | 99.17 | 95.00 | 3.41 | 7.47 | 7.67 |  | 94.94 | 4.74 | 5.68 | 7.00 | GWAS |
| NSFTV321 | AUS | DM 56 | Bangladesh | 99.50 | 95.00 | 3.76 | 6.94 | 7.67 |  | 89.00 | 6.24 | 4.39 | 5.67 | GWAS |
| NSFTV325 | IND | EMATA A 16-34 | Myanmar | 100.00 | 100.00 | 2.67 | 9.76 | 8.67 |  | 100.00 | 2.37 | 10.10 | 8.67 | GWAS |
| NSFTV326 | AUS | Ghorbhai | Bangladesh | 100.00 | 95.00 | 4.99 | 5.37 | 7.00 |  | 92.00 | 4.51 | 5.86 | 7.00 | GWAS |
| NSFTV328 | AUS | Jamir | Bangladesh | 98.33 | 98.33 | 3.27 | 7.64 | 8.00 |  | 100.00 | 2.72 | 9.61 | 8.67 | GWAS |
| NSFTV332 | ADMIX | KPF-16 | Bangladesh | 99.17 | 100.00 | 2.74 | 9.75 | 8.67 |  | 98.33 | 2.16 | 10.13 | 8.67 | GWAS |
| NSFTV334 | TEJ | Lomello | Thailand | 96.74 | 80.00 | 5.42 | 4.22 | 5.67 |  | 91.75 | 5.33 | 5.01 | 6.67 | GWAS |
| NSFTV337 | IND | Sabharaj | Bangladesh | 98.47 | 95.00 | 2.87 | 8.45 | 8.33 |  | 98.81 | 3.16 | 8.82 | 8.00 | GWAS |
| NSFTV340 | ADMIX | Berenj | Afghanistan | 99.17 | 100.00 | 2.45 | 10.27 | 8.67 |  | 98.33 | 2.70 | 9.74 | 8.67 | GWAS |
| NSFTV342 | TRJ | Cenit | Argentina | 98.00 | 95.00 | 2.35 | 10.02 | 8.67 |  | 97.98 | 3.16 | 8.34 | 8.00 | GWAS |
| NSFTV345 | AUS | DZ 193 | Bangladesh | 97.13 | 91.67 | 4.26 | 5.88 | 7.00 |  | 63.67 | 8.11 | 2.77 | 4.00 | GWAS |
| NSFTV346 | AUS | Karkati 87 | Bangladesh | 100.00 | 91.67 | 3.14 | 7.71 | 8.00 |  | 98.28 | 4.28 | 6.75 | 7.33 | GWAS |
| NSFTV347 | TRJ | Creole | Belize | 100.00 | 95.00 | 3.59 | 7.14 | 7.67 |  | 98.33 | 4.11 | 7.28 | 7.33 | GWAS |
| NSFTV348 | IND | China 1039 | China | 98.33 | 96.67 | 3.56 | 7.31 | 7.67 |  | 97.96 | 3.29 | 7.84 | 8.00 | GWAS |
| NSFTV350 | TRJ | Ligerito | Colombia | 98.50 | 100.00 | 3.34 | 7.69 | 8.00 |  | 98.98 | 3.11 | 8.53 | 8.00 | GWAS |
| NSFTV352 | TRJ | Guatemala 1021 | Guatemala | 97.33 | 100.00 | 2.59 | 9.88 | 8.67 |  | 99.00 | 3.18 | 8.23 | 8.00 | GWAS |
| NSFTV355 | TEJ | ASD 1 | India | 96.23 | 73.33 | 6.85 | 3.56 | 5.33 |  | 96.43 | 4.44 | 6.30 | 7.33 | GWAS |
| NSFTV357 | AUS | 9524.00 | India | 95.17 | 91.67 | 4.64 | 5.35 | 7.00 |  | 90.82 | 4.75 | 5.47 | 7.00 | GWAS |
| NSFTV360 | AUS | PTB 30 | India | 95.98 | 90.00 | 4.61 | 5.14 | 6.67 |  | 73.00 | 7.44 | 3.42 | 5.00 | GWAS |
| NSFTV364 | ADMIX | Rikuto Norin 21 | Japan | 96.67 | 93.33 | 3.42 | 7.18 | 7.67 |  | 95.00 | 4.95 | 5.14 | 7.00 | GWAS |
| NSFTV365 | TEJ | Shirogane | Japan | 95.83 | 96.67 | 2.96 | 8.55 | 8.33 |  | 98.15 | 3.21 | 8.78 | 8.00 | GWAS |
| NSFTV372 | AUS | Sufaid | Pakistan | 99.17 | 98.33 | 3.54 | 7.50 | 7.67 |  | 96.67 | 4.60 | 6.25 | 7.33 | GWAS |
| NSFTV375 | TRJ | Upland | PONAPE ISLAND | 99.17 | 95.00 | 3.28 | 7.63 | 8.00 |  | 100.00 | 4.09 | 7.20 | 7.33 | GWAS |
| NSFTV376 | ADMIX | Breviaristata | Portugal | 99.17 | 96.67 | 3.36 | 7.66 | 8.00 |  | 98.28 | 5.18 | 5.54 | 6.67 | GWAS |
| NSFTV377 | TRJ | PR 304 | Puerto Rico | 100.00 | 98.33 | 2.76 | 9.37 | 8.67 |  | 96.61 | 4.01 | 7.02 | 7.33 | GWAS |
| NSFTV384 | TRJ | 318.00 | TURKEY | 99.48 | 96.67 | 3.45 | 7.50 | 7.67 |  | 99.00 | 4.34 | 6.22 | 7.33 | GWAS |
| NSFTV386 | ADMIX | Palmyra | United States | 99.17 | 95.00 | 1.89 | 13.13 | 9.67 |  | 98.33 | 2.73 | 9.39 | 8.67 | GWAS |
| NSFTV388 | ADMIX | Nortai | United States | 99.17 | 96.67 | 3.05 | 8.26 | 8.00 |  | 93.33 | 4.35 | 6.31 | 7.33 | GWAS |
| NSFTV392 | TRJ | Edith | United States | 98.33 | 98.33 | 3.13 | 8.20 | 8.00 |  | 98.33 | 3.88 | 7.59 | 8.00 | GWAS |
| NSFTV394 | TRJ | Lady Wright Seln | United States | 99.50 | 98.33 | 2.66 | 9.50 | 8.67 |  | 95.96 | 3.74 | 6.97 | 7.67 | GWAS |
| NSFTV397 | TRJ | Cybonnet | United States | 97.00 | 96.67 | 2.77 | 9.06 | 8.67 |  | 99.00 | 2.99 | 8.59 | 8.33 | GWAS |
| NSFTV621 | TRJ | LaGrue | United States | 99.17 | 100.00 | 2.66 | 9.56 | 8.67 |  | 96.97 | 3.02 | 8.80 | 8.00 | GWAS |
| NSFTV622 | ADMIX | Bengal | United States | 97.50 | 91.67 | 3.18 | 7.67 | 8.00 |  | 100.00 | 3.72 | 7.99 | 8.00 | GWAS |
| NSFTV624 | TRJ | Kaybonnet | United States | 100.00 | 96.67 | 3.11 | 8.02 | 8.00 |  | 98.98 | 3.18 | 8.15 | 8.00 | GWAS |
| NSFTV625 | TRJ | Katy | United States | 96.36 | 100.00 | 3.04 | 8.48 | 8.00 |  | 94.12 | 4.66 | 5.96 | 7.00 | GWAS |
| NSFTV626 | IND | C101A51 | Colombia | 99.17 | 98.08 | 2.76 | 9.29 | 8.67 |  | 100.00 | 3.23 | 8.87 | 8.00 | GWAS |
| NSFTV628 | TRJ | Jefferson | United States | 98.33 | 96.67 | 3.48 | 7.32 | 7.67 |  | 96.67 | 4.08 | 7.12 | 7.33 | GWAS |
| NSFTV630 | TRJ | Saber | United States | 98.67 | 100.00 | 2.67 | 9.78 | 8.67 |  | 97.00 | 3.34 | 7.59 | 8.00 | GWAS |
| NSFTV633 | IND | Jing 185-7 | China | 99.50 | 90.00 | 5.64 | 4.70 | 6.33 |  | 95.83 | 5.37 | 5.51 | 6.67 | GWAS |
| NSFTV634 | IND | Rondo (4484-1693) | China | 98.99 | 98.33 | 4.10 | 6.37 | 7.33 |  | 94.00 | 4.52 | 5.79 | 7.00 | GWAS |
| NSFTV641 | TEJ | Tainung 67 | Taiwan | 98.30 | 95.00 | 3.64 | 6.81 | 7.67 |  | 96.30 | 5.27 | 5.11 | 6.67 | GWAS |
| NSFTV644 | IND | IR64 | Philippines | 97.67 | 95.00 | 3.72 | 6.90 | 7.67 |  | 97.96 | 3.96 | 6.98 | 7.67 | GWAS |
| NSFTV647 | TRJ | Cypress | United States | 97.83 | 98.33 | 3.28 | 7.64 | 8.00 |  | 98.98 | 3.28 | 7.92 | 8.00 | GWAS |
| NSFTV40 | ADMIX | Dam | Thailand | 90.00 | \ | \ | \ | \ |  | \ | \ | \ | \ | \ |
| NSFTV56 | TEJ | Geumobyeo | South Korea | 87.68 | \ | \ | \ | \ |  | \ | \ | \ | \ | \ |
| NSFTV67 | TEJ | Hu Lo Tao | China | 91.67 | \ | \ | \ | \ |  | \ | \ | \ | \ | \ |
| NSFTV101 | TRJ | Lemont | United States | 82.00 | \ | \ | \ | \ |  | \ | \ | \ | \ | \ |
| NSFTV107 | TRJ | NSF-TV 107 | Bangladesh | 88.33 | \ | \ | \ | \ |  | \ | \ | \ | \ | \ |
| NSFTV147 | TRJ | Sinampaga Selection | Philippines | 93.33 | \ | \ | \ | \ |  | \ | \ | \ | \ | \ |
| NSFTV163 | IND | Taducan | Philippines | 91.67 | \ | \ | \ | \ |  | \ | \ | \ | \ | \ |
| NSFTV164 | TRJ | Tondok | Indonesia | 91.67 | \ | \ | \ | \ |  | \ | \ | \ | \ | \ |
| NSFTV169 | TEJ | WC 6 | China | 91.50 | \ | \ | \ | \ |  | \ | \ | \ | \ | \ |
| NSFTV204 | TEJ | Razza 77 | Italy | 92.00 | \ | \ | \ | \ |  | \ | \ | \ | \ | \ |
| NSFTV217 | ADMIX | YRL-1 | Australia | 93.16 | \ | \ | \ | \ |  | \ | \ | \ | \ | \ |
| NSFTV219 | TEJ | Nucleoryza | Austria | 92.66 | \ | \ | \ | \ |  | \ | \ | \ | \ | \ |
| NSFTV225 | TEJ | Biser 1 | Bulgaria | 85.82 | \ | \ | \ | \ |  | \ | \ | \ | \ | \ |
| NSFTV244 | ADMIX | Arabi | Egypt | 88.33 | \ | \ | \ | \ |  | \ | \ | \ | \ | \ |
| NSFTV248 | TEJ | Caucasica | Former Soviet Union | 87.81 | \ | \ | \ | \ |  | \ | \ | \ | \ | \ |
| NSFTV249 | ADMIX | Pirinae 69 | Former Yugoslavia | 79.71 | \ | \ | \ | \ |  | \ | \ | \ | \ | \ |
| NSFTV250 | TEJ | Bulgare | France | 81.10 | \ | \ | \ | \ |  | \ | \ | \ | \ | \ |
| NSFTV256 | TEJ | Romanica | Hungary | 79.44 | \ | \ | \ | \ |  | \ | \ | \ | \ | \ |
| NSFTV258 | TRJ | Tia Bura | Indonesia | 93.33 | \ | \ | \ | \ |  | \ | \ | \ | \ | \ |
| NSFTV265 | TEJ | Vialone | Italy | 91.67 | \ | \ | \ | \ |  | \ | \ | \ | \ | \ |
| NSFTV270 | ADMIX | Osogovka | Macedonia | 82.38 | \ | \ | \ | \ |  | \ | \ | \ | \ | \ |
| NSFTV272 | ADMIX | 923.00 | Madagascar | 89.58 | \ | \ | \ | \ |  | \ | \ | \ | \ | \ |
| NSFTV275 | TEJ | Sri Malaysia Dua | Malaysia | 92.86 | \ | \ | \ | \ |  | \ | \ | \ | \ | \ |
| NSFTV280 | ADMIX | Saku | Mongolia | 93.33 | \ | \ | \ | \ |  | \ | \ | \ | \ | \ |
| NSFTV286 | TRJ | IITA 135 | Nigeria | 86.67 | \ | \ | \ | \ |  | \ | \ | \ | \ | \ |
| NSFTV296 | TEJ | Dosel | Spain | 93.19 | \ | \ | \ | \ |  | \ | \ | \ | \ | \ |
| NSFTV299 | IND | SML 242 | Suriname | 93.13 | \ | \ | \ | \ |  | \ | \ | \ | \ | \ |
| NSFTV370 | AUS | Coarse | Pakistan | 74.17 | \ | \ | \ | \ |  | \ | \ | \ | \ | \ |
| NSFTV373 | AROMATIC | Lambayeque 1 | Peru | 92.50 | \ | \ | \ | \ |  | \ | \ | \ | \ | \ |
| NSFTV378 | AUS | Kalubala Vee | Sri Lanka | 71.50 | \ | \ | \ | \ |  | \ | \ | \ | \ | \ |
| NSFTV616 | IND | RT0034 | United States | 89.74 | \ | \ | \ | \ |  | \ | \ | \ | \ | \ |
| NSFTV651 | AUS | Dular | India | 90.00 | \ | \ | \ | \ |  | \ | \ | \ | \ | \ |

**Table S2** Primer pairs used in this study.

| Primer names | Primer sequences (5’–3’) | Use |
| --- | --- | --- |
| *OsActin*-F | AGGAAGGCTGGAAGAGGACC | qRT-PCR |
| *OsActin*-R | CGGGAAATTGTGAGGGACAT |
| *OsTTL*-F | TCACCTAGAGATGTGGAAGG |
| *OsTTL*-R | TTGTTCGTAACCGATGAGC |
| *OsSAPK1*-F | CCAGACTACGTTCGAGTTTCAG |
| *OsSAPK1*-R | GTGGTTCTTGATCTCTGGGATAG |
| *OsTTL*-BsF | AATAATGGTCTCAGGCGAGGACGTGCTGCGCGTGAA | CRISPR vector construction |
| *OsTTL*-F0 | GAGGACGTGCTGCGCGTGAAGTTTTAGAGCTAGAAATAGC |
| *OsTTL*-BsR | ATTATTGGTCTCTAAACATGGCCGGGTGCGCGGCGA |
| *OsTTL*-R0 | ATGGCCGGGTGCGCGGCGACGCTTCTTGGTGCC |
| *OsSAPK1*-BsF | AATAATGGTCTCAGGCGGATGAGGGACATCGGGTCC |
| *OsSAPK1*-F0 | GGATGAGGGACATCGGGTCCGTTTTAGAGCTAGAAATAGC |
| *OsSAPK1*-BsR | ATTATTGGTCTCTAAACTGAGTCCCCTCTCGATGAA |
| *OsSAPK1*-R0 | TGAGTCCCCTCTCGATGAACGCTTCTTGGTGCC |
| OsU3-FD3 | GACAGGCGTCTTCTACTGGTGCTAC | Bacteria PCR |
| TaU3-RD | CTCACAAATTATCAGCACGCTAGTC |
| TaU3-FD2 | TTGACTAGCGTGCTGATAATTTGTG |
| *OsTTL*-CRISPR-F | CTTGAGAATAGAAGCCCACA | Sequencing |
| *OsTTL*-CRISPR-R | AACTGATAACGCCGCAGA |
| *OsSAPK1*-CRISPR-F | GCCGCCGCCTATACAAAG |
| *OsSAPK1*-CRISPR-R | AACTAACGCTAACCGATTCTC |

**Table S3** Details of the significant loci for GR, *T_50_*, GI and ML under salt stress in 2015 and 2017 years.

| Year | Locus | Traits | Chr. | Subgroups | Lead SNP ID. | Alleles | *P*-value | Lead SNP position (bp) | Position range (bp) | Known QTLs |
| --- | --- | --- | --- | --- | --- | --- | --- | --- | --- | --- |
| 2015 | *qNL1.1* | GR | 1 | *INDICA* | SNP-1.7526310. | G/A | 1.26E-08 | 7527311 | 7427311 ~ 7627311 | *OsNLP3* (Yi et al. 2022) |
|  |  | *T_50_* | 1 | *INDICA* | SNP-1.7526310. | G/A | 6.34E-08 |
|  | *qNL1.2* | *T_50_* | 1 | *All* | SNP-1.12473321. | G/A | 4.37E-06 | 12474348 | 12374348 ~ 12574348 |  |
|  | *qNL1.3* | GR | 1 | *INDICA* | SNP-1.21276716. | C/G | 5.57E-10 | 21277762 | 21177762 ~ 21377762 |  |
|  |  | *T_50_* | 1 | *INDICA* | SNP-1.21276716. | C/G | 1.64E-10 |  |
|  |  | ML | 1 | *INDICA* | SNP-1.21276716. | C/G | 3.84E-10 |  |
|  | *qNL1.4* | GR | 1 | *INDICA* | SNP-1.21942868. | A/G | 1.26E-14 | 21943914 | 21843914 ~ 22043914 |  |
|  |  | *T_50_* | 1 | *INDICA* | SNP-1.21942868. | A/G | 1.73E-13 |  |
|  |  | ML | 1 | *INDICA* | SNP-1.21942868. | A/G | 2.48E-11 |  |
|  | *qNL1.5* | GR | 1 | *INDICA* | SNP-1.22524873. | C/T | 5.77E-10 | 22525919 | 22425919 ~ 22625919 |  |
|  |  | *T_50_* | 1 | *INDICA* | SNP-1.22524873. | C/T | 2.33E-10 |  |
|  |  | ML | 1 | *INDICA* | SNP-1.22524873. | C/T | 2.74E-09 |  |
|  | *qNL1.6* | GR | 1 | *INDICA* | SNP-1.36008253. | C/T | 4.94E-08 | 36009297 | 35909297 ~ 36109297 | *OsMYB3R-2* (Dai et al. 2007); *ZFP179* (Sun et al. 2010) |
|  |  | *T_50_* | 1 | *INDICA* | SNP-1.36008253. | C/T | 1.15E-07 |
|  | *qNL1.7* | GR | 1 | *INDICA* | SNP-1.40704195. | C/G | 3.36E-10 | 40705239 | 40605239 ~ 40805239 |  |
|  |  | *T_50_* | 1 | *INDICA* | SNP-1.40704195. | C/G | 5.47E-10 |  |
|  | *qNL1.8* | GR | 1 | *INDICA* | SNP-1.41956360. | G/A | 8.22E-12 | 41957404 | 41857404 ~ 42057404 |  |
|  |  | *T_50_* | 1 | *INDICA* | SNP-1.41956360. | G/A | 2.26E-11 |  |
|  | *qNL2.1* | GR | 2 | *INDICA* | SNP-2.15726489. | G/A | 3.65E-10 | 15732360 | 15632360 ~ 15832360 |  |
|  | *qNL2.2* | GR | 2 | *INDICA* | SNP-2.20568287. | G/A | 2.00E-08 | 20574156 | 20474156 ~ 20674156 |  |
|  | *qNL3.1* | GI | 3 | *INDICA* | SNP-3.15633835. | C/A | 8.53E-06 | 15635189 | 15508568 ~ 15809610 | *qLTG-3* (Jiang et al. 2006); *OsSAPK1* (Lou et al. 2018) |
|  |  |  |  |  | SNP-3.15708256. | G/A | 8.53E-06 | 15709610 |
|  |  | *T_50_* | 3 | *INDICA* | SNP-3.15607214. | T/C | 6.71E-09 | 15608568 |
|  |  | ML | 3 | *INDICA* | SNP-3.15633835. | C/A | 1.23E-08 | 15635189 |
|  |  |  |  |  | SNP-3.15708256. | G/A | 1.23E-08 | 15709610 |
|  | *qNL3.2* | GR | 3 | *INDICA* | SNP-3.16475982. | G/A | 2.32E-15 | 16477338 | 16197591 ~ 16577338 |  |
|  |  | GI | 3 | *INDICA* | SNP-3.16296237. | C/T | 2.21E-06 | 16297591 |  |
|  |  | *T_50_* | 3 | *INDICA* | SNP-3.16475982. | G/A | 1.23E-15 | 16477338 |  |
|  |  | ML | 3 | *INDICA* | SNP-3.16475982. | G/A | 1.37E-12 |  |
|  | *qNL3.3* | GR | 3 | *INDICA* | SNP-3.28589583. | G/A | 2.15E-14 | 28596531 | 28496531 ~ 28696531 | *qGR3-1* (Cui et al. 2002) |
|  | *qNL4.1* | GR | 4 | *INDICA* | SNP-4.25541697. | G/A | 2.55E-11 | 25726836 | 25520731 ~ 26006194 | *qGR-3d4* (Nakhla et al. 2021) |
|  |  | *T_50_* | 4 | *INDICA* | SNP-4.25435591. | C/G | 1.72E-11 | 25620731 |
|  |  | ML | 4 | *INDICA* | SNP-4.25721068. | C/T | 1.37E-09 | 25906194 |
|  | *qNL5.1* | GR | 5 | *INDICA* | SNP-5.2314035. | C/T | 9.02E-09 | 2314061 | 2214061 ~ 2414061 |  |
|  |  | ML | 5 | *INDICA* | SNP-5.2314035. | C/T | 3.19E-08 |  |
|  |  | *T_50_* | 5 | *INDICA* | SNP-5.2314035. | C/T | 3.09E-09 |  |
|  | *qNL5.2* | GR | 5 | *INDICA* | SNP-5.3162251. | T/A | 8.86E-16 | 3162276 | 3062276 ~ 3262276 |  |
|  |  | *T_50_* | 5 | *INDICA* | SNP-5.3162251. | T/A | 4.60E-13 |  |
|  |  | ML | 5 | *INDICA* | SNP-5.3162251. | T/A | 2.06E-11 |  |
|  | *qNL5.3* | *T_50_* | 5 | *INDICA* | SNP-5.27624340. | A/T | 3.95E-09 | 27686984 | 27586984 ~ 27786984 | *qSV‐5c* (Xie et al. 2014) |
|  |  | ML | 5 | *INDICA* | SNP-5.27624340. | A/T | 1.41E-08 |
|  | *qNL6.1* | *T_50_* | 6 | *INDICA* | SNP-6.361762. | C/T | 2.86E-09 | 362763 | 262763 ~ 514288 |  |
|  |  | ML | 6 | *INDICA* | SNP-6.361762. | C/T | 9.85E-08 |  |
|  |  | GR | 6 | *INDICA* | SNP-6.413287. | A/G | 4.42E-08 | 414288 |  |
|  | *qNL6.2* | GR | 6 | *INDICA* | SNP-6.1267518. | G/A | 2.87E-10 | 1268518 | 1168518 ~ 1368518 | SSI_IR_48h_rs6_1459330 (Cui et al. 2018) |
|  | *qNL6.3* | GR | 6 | *INDICA* | SNP-6.25948893. | C/T | 2.28E-08 | 25949891 | 25849891 ~ 26049891 | *OsSAE1* (Li et al. 2022); *qRI6* (Islam et al. 2022) |
|  | *qNL7.1* | GR | 7 | *INDICA* | SNP-7.1127433. | C/A | 1.05E-10 | 1128434 | 1028434 ~ 1228434 |  |
|  |  | *T_50_* | 7 | *INDICA* | SNP-7.1127433. | C/A | 1.05E-10 |  |
|  | *qNL7.2* | GR | 7 | *INDICA* | SNP-7.12667704. | C/T | 7.80E-08 | 12668698 | 12568698 ~ 12768698 |  |
|  | *qNL7.3* | GR | 7 | *INDICA* | SNP-7.22877864. | G/A | 7.37E-10 | 22878858 | 22778858 ~ 22978858 |  |
|  | *qNL7.4* | GR | 7 | *INDICA* | SNP-7.26091453. | G/A | 3.45E-08 | 26092448 | 25992448 ~ 26192448 |  |
|  | *qNL8.1* | GR | 8 | *INDICA* | SNP-8.2372428. | A/G | 4.85E-08 | 2373426 | 2273426 ~ 2473426 |  |
|  |  | *T_50_* | 8 | *INDICA* | SNP-8.2372428. | A/G | 2.91E-10 |  |
|  |  | ML | 8 | *INDICA* | SNP-8.2372428. | A/G | 1.22E-08 |  |
|  | *qNL8.2* | *T_50_* | 8 | *All* | SNP-8.5186188. | A/G | 3.97E-07 | 5187186 | 5087186 ~ 5531847 |  |
|  |  | GI | 8 | *All* | SNP-8.5369331. | G/T | 1.54E-06 | 5370329 |  |
|  |  | *T_50_* | 8 | *JAPONICA* | SNP-8.5369331. | G/T | 1.45E-08 |  |
|  |  | ML | 8 | *JAPONICA* | SNP-8.5430849. | A/G | 7.55E-07 | 5431847 |  |
|  | *qNL10.1* | GR | 10 | *INDICA* | SNP-10.675933. | A/T | 1.98E-08 | 676958 | 576958 ~ 776958 |  |
|  | *qNL11.1* | GR | 11 | *JAPONICA* | SNP-11.2921485. | C/T | 9.38E-09 | 2925582 | 2788595 ~ 3025582 |  |
|  |  | *T_50_* | 11 | *JAPONICA* | SNP-11.2884498. | C/T | 3.02E-08 | 2888595 |  |
|  |  | ML | 11 | *JAPONICA* | SNP-11.2884498. | C/T | 1.70E-07 |  |
|  | *qNL11.2* | *T_50_* | 11 | *All* | SNP-11.10736817. | G/A | 5.93E-06 | 10742273 | 10582418 ~ 10842273 |  |
|  |  |  |  | *INDICA* | SNP-11.10676962. | A/T | 4.46E-13 | 10682418 |  |
|  | *qNL11.3* | GR | 11 | *All* | SNP-11.15410183. | G/T | 8.85E-08 | 15875313 | 15775313 ~ 15975313 | *Rab16A* (Ganguly et al. 2012) |
|  |  | *T_50_* | 11 | *All* | SNP-11.15410183. | G/T | 8.24E-10 |
|  | *qNL11.4* | GR | 11 | *INDICA* | SNP-11.15993272. | T/C | 2.83E-09 | 16458396 | 16358396 ~ 16558396 |  |
|  | *qNL12.1* | GR | 12 | *INDICA* | SNP-12.2919955. | G/A | 1.84E-11 | 2920954 | 2820954 ~ 3020954 | *qGR-3d12* (Nakhla et al. 2021) |
|  |  | *T_50_* | 12 | *INDICA* | SNP-12.2919955. | G/A | 4.17E-11 |
|  |  | ML | 12 | *INDICA* | SNP-12.2919955. | G/A | 5.63E-09 |
|  | *qNL12.2* | GR | 12 | *INDICA* | SNP-12.16362757. | T/A | 1.82E-09 | 16365462 | 16265462 ~ 16465462 | chr12_1628276 (Yu et al. 2018) |
|  |  | *T_50_* | 12 | *INDICA* | SNP-12.16362757. | T/A | 8.54E-09 |
|  | *qNL12.3* | GR | 12 | *INDICA* | SNP-12.19954018. | C/A | 1.11E-11 | 19982577 | 19836014 ~ 20082577 |  |
|  |  | *T_50_* | 12 | *INDICA* | SNP-12.19907483. | C/A | 1.79E-10 | 19936014 |  |
|  |  | ML | 12 | *INDICA* | SNP-12.19907483. | C/A | 4.78E-09 |  |
|  | *qNL12.4* | GR | 12 | *INDICA* | SNP-12.20834996. | C/G | 1.42E-11 | 20868448 | 20768448 ~ 20968448 |  |
|  |  | *T_50_* | 12 | *INDICA* | SNP-12.20834996. | C/G | 2.07E-09 |  |
|  |  | ML | 12 | *INDICA* | SNP-12.20834996. | C/G | 1.13E-08 |  |
| 2017 | *qNL1.9* | ML | 1 | *JAPONICA* | SNP-1.731067. | C/T | 1.77E-06 | 732068 | 632068 ~ 832068 |  |
|  | *qNL1.10* | GR | 1 | *All* | SNP-1.41156269. | T/C | 4.75E-09 | 41157313 | 41050961 ~ 41257313 |  |
|  |  |  |  | *INDICA* | SNP-1.41156269. | T/C | 1.19E-07 |  |
|  |  | GI | 1 | *INDICA* | SNP-1.41149917. | C/T | 8.86E-06 | 41150961 |  |
|  |  | *T_50_* | 1 | *All* | SNP-1.41156269. | T/C | 2.39E-08 | 41157313 |  |
|  |  | ML | 1 | *All* | SNP-1.41156269. | T/C | 3.63E-09 | 41157313 |  |
|  |  |  |  | *INDICA* | SNP-1.41149917. | C/T | 3.92E-07 | 41150961 |  |
|  | *qNL1.11* | ML | 1 | *INDICA* | SNP-1.41603495. | T/G | 5.46E-07 | 41604539 | 41504539 ~ 41704539 |  |
|  | *qNL1.8* | GR | 1 | *INDICA* | SNP-1.41956360. | G/A | 3.38E-08 | 41957404 | 41857404 ~ 42057404 |  |
|  | *qNL3.1* | GR | 3 | *All* | SNP-3.15846851. | C/T | 1.20E-06 | 15848205 | 15508568 ~ 15999652 | *qLTG-3* (Jiang et al. 2006), *OsSAPK1* (Lou et al. 2018) |
|  |  |  |  | *INDICA* | SNP-3.15607214. | T/C | 1.63E-08 | 15608568 |
|  |  | *T_50_* | 3 | *All* | SNP-3.15760902. | G/A | 1.54E-07 | 15762256 |
|  |  |  |  | *INDICA* | SNP-3.15607214. | T/C | 6.23E-09 | 15608568 |
|  |  | ML | 3 | *All* | SNP-3.15898298. | C/T | 1.17E-06 | 15899652 |
|  |  |  |  | *INDICA* | SNP-3.15607214. | T/C | 3.32E-07 | 15608568 |
|  | *qNL3.2* | GR | 3 | *INDICA* | SNP-3.16475982. | G/A | 2.31E-09 | 16477338 | 16377338 ~ 16577338 |  |
|  |  | *T_50_* | 3 | *INDICA* | SNP-3.16475982. | G/A | 1.60E-08 |  |
|  | *qNL4.2* | GR | 4 | *JAPONICA* | SNP-4.377700. | G/A | 4.09E-10 | 378703 | 278703 ~ 478703 | *qGR4* (Naveed et al. 2018) |
|  | *qNL4.1* | GR | 4 | *INDICA* | SNP-4.25487271. | C/T | 2.58E-08 | 25672410 | 25572410 ~ 25862457 | *qGR-3d4* (Nakhla et al. 2021) |
|  |  | *T_50_* | 4 | *INDICA* | SNP-4.25577318. | T/C | 5.52E-08 | 25762457 |
|  | *qNL5.4* | ML | 5 | *INDICA* | SNP-5.7014353. | G/A | 1.15E-06 | 7014412 | 6914412 ~ 7128248 | *qSSD5*_*qSST5* (Naveed et al. 2018) |
|  |  | GR | 5 | *All* | SNP-5.7016625. | C/T | 1.16E-06 | 7016684 |
|  |  | *T_50_* | 5 | *All* | SNP-5.7016625. | C/T | 2.30E-08 | 7016684 |
|  | *qNL7.5* | GR | 7 | *JAPONICA* | SNP-7.6733367. | C/T | 1.48E-13 | 6734363 | 6634363 ~ 6834363 |  |
|  | *qNL7.6* | GR | 7 | *All* | SNP-7.9823128. | T/A | 3.72E-09 | 9824123 | 9724123 ~ 10063364 |  |
|  |  | GR | 7 | *JAPONICA* | SNP-7.9962369. | A/T | 7.53E-08 | 9963364 |  |
|  | *qNL7.2* | GR | 7 | *All* | SNP-7.12697563. | G/A | 9.52E-08 | 12698557 | 12598557 ~ 12798557 |  |
|  | *qNL7.7* | GR | 7 | *INDICA* | SNP-7.13252344. | T/C | 7.28E-10 | 13253338 | 13153338 ~ 13353338 |  |
|  | *qNL7.8* | GR | 7 | *INDICA* | SNP-7.13671383. | A/T | 8.51E-08 | 13672377 | 13572377 ~ 13772377 |  |
|  | *qNL7.9* | GR | 7 | *All* | SNP-7.17902946. | G/A | 4.90E-06 | 17903940 | 17803940 ~ 18003940 |  |
|  | *qNL7.4* | GR | 7 | *All* | SNP-7.26165263. | C/T | 2.03E-08 | 26166258 | 26066258 ~ 26266258 |  |
|  | *qNL9.1* | ML | 9 | *JAPONICA* | SNP-9.11517994. | A/G | 9.30E-07 | 11518996 | 11418996 ~ 11618996 | *SSI_MGT_rs9_11450011* (Cui et al. 2018) |
|  | *qNL10.2* | GR | 10 | *JAPONICA* | SNP-10.4700118. | G/T | 1.55E-11 | 4683582 | 4583582 ~ 4783582 | *qGR-7d10* (Nakhla et al. 2021) |
|  |  | ML | 10 | *JAPONICA* | SNP-10.4700118. | G/T | 1.51E-09 |
|  |  | *T_50_* | 10 | *JAPONICA* | SNP-10.4700118. | G/T | 3.91E-08 |
|  | *qNL11.4* | GR | 11 | *All* | SNP-11.15816639. | T/C | 2.55E-07 | 16281767 | 16181767 ~ 16381767 |  |
|  | *qNL11.5* | GR | 11 | *All* | SNP-11.17793041. | T/A | 4.59E-06 | 18259183 | 18159183 ~ 18359183 |  |
|  |  | ML | 11 | *All* | SNP-11.17793041. | T/A | 6.78E-06 |  |
|  | *qNL11.6* | GR | 11 | *All* | SNP-11.21533545. | G/T | 9.38E-07 | 21999675 | 21899675 ~ 22099675 | *qSV-11* (Xie et al. 2014) |

**Table S4** Candidate genes of the key locus *qNL3.1* associated with seed germination under salt stress.

| Gene ID. | Annotation |
| --- | --- |
| LOC_Os03g27090 | MYB family transcription factor, putative, expressed |
| LOC_Os03g27110 | hydrolase protein, putative, expressed |
| LOC_Os03g27120 | ICE-like protease p20 domain containing protein, putative, expressed |
| LOC_Os03g27160 | expressed protein |
| LOC_Os03g27170 | ICE-like protease p20 domain containing protein, putative, expressed |
| LOC_Os03g27190 | ICE-like protease p20 domain containing protein, putative, expressed |
| LOC_Os03g27200 | expressed protein |
| LOC_Os03g27210 | LOL3, putative, expressed |
| LOC_Os03g27230 | phospho-2-dehydro-3-deoxyheptonate aldolase, chloroplast precursor, putative, expressed |
| LOC_Os03g27250 | OsFBO14 - F-box and other domain containing protein, expressed |
| LOC_Os03g27260 | 40S ribosomal protein S6, putative, expressed |
| **LOC_Os03g27280** | CAMK_CAMK_like.19 - CAMK includes calcium/calmodulin depedent protein kinases, expressed |
| LOC_Os03g27290 | cytochrome c oxidase subunit, putative, expressed |
| LOC_Os03g27300 | hypothetical protein |
| LOC_Os03g27310 | histone H3, putative, expressed |
| **LOC_Os03g27320** | steroid binding protein, putative, expressed |
| LOC_Os03g27340 | expressed protein |
| LOC_Os03g27350 | cysteine protease ATG4, putative, expressed |
| LOC_Os03g27360 | RING-H2 finger protein ATL5H precursor, putative, expressed |
| LOC_Os03g27370 | phospholipase D, putative, expressed |
| LOC_Os03g27380 | hypothetical protein |
| LOC_Os03g27390 | CPuORF35 - conserved peptide uORF-containing transcript, expressed |
| LOC_Os03g27410 | expressed protein |
| LOC_Os03g27430 | expressed protein |
| LOC_Os03g27440 | hypothetical protein |
| LOC_Os03g27450 | ADP-ribosylation factor, putative, expressed |
| LOC_Os03g27460 | heat shock protein DnaJ, putative, expressed |
| LOC_Os03g27470 | hypothetical protein |
| LOC_Os03g27480 | OsSCP14 - Putative Serine Carboxypeptidase homologue, expressed |
| LOC_Os03g27490 | expressed protein |
| LOC_Os03g27510 | OsSCP15 - Putative Serine Carboxypeptidase homologue, expressed |
| LOC_Os03g27530 | OsSCP16 - Putative Serine Carboxypeptidase homologue, expressed |
| LOC_Os03g27550 | OsSCP17 - Putative Serine Carboxypeptidase homologue, expressed |
| LOC_Os03g27560 | expressed protein |
| LOC_Os03g27570 | expressed protein |
| LOC_Os03g27580 | expressed protein |
| LOC_Os03g27590 | OsSCP18 - Putative Serine Carboxypeptidase homologue, expressed |
| LOC_Os03g27610 | patatin, putative, expressed |
| LOC_Os03g27680 | conserved hypothetical protein |
| LOC_Os03g27690 | expressed protein |
| LOC_Os03g27700 | expressed protein |
| LOC_Os03g27740 | expressed protein |
| LOC_Os03g27750 | expressed protein |
| LOC_Os03g27760 | nodulation protein-related, putative, expressed |
| LOC_Os03g27770 | heme oxygenase 2, putative, expressed |
| LOC_Os03g27780 | protein phosphotase protein, putative, expressed |
| LOC_Os03g27790 | EF hand family protein, putative |
| LOC_Os03g27800 | paramyosin, putative, expressed |
| LOC_Os03g27820 | expressed protein |
| LOC_Os03g27830 | expressed protein |
| LOC_Os03g27840 | splicing factor, arginine/serine-rich 16, putative, expressed |

**Table S5** Significant SNP analysis in the candidate region of *qNL3.1*.

| Groups | Functional SNPs ID. | Physical position (bp) | Alleles | SNP location | Gene ID. | Functional annotation |
| --- | --- | --- | --- | --- | --- | --- |
| Group I | SNP-3.15650453. | 15651807 | A/T | Lys309Asn | LOC_Os03g27320 | steroid binding protein, putative, expressed |
| Group III | SNP-3.15612217. | 15613571 | T/C | Promotor | LOC_Os03g27250 | OsFBO14 - F-box and other domain containing protein, expressed |
|  | SNP-3.15631187. | 15632541 | C/T | Promotor | LOC_Os03g27280 | CAMK_CAMK_like.19 - CAMK includes calcium/calmodulin depedent protein kinases, expressed |
|  | SNP-3.15645395. | 15646749 | G/A | Promotor | LOC_Os03g27310 | histone H3, putative, expressed |
|  | SNP-3.15665224. | 15666578 | C/T | Promotor | LOC_Os03g27360 | RING-H2 finger protein ATL5H precursor, putative, expressed |
|  | SNP-3.15665816. | 15667170 | A/G | Promotor |  |  |
